# Molecular Compasses for Modulating Electronic Communication in Pillar[5]quinone

**DOI:** 10.1021/jacs.4c12280

**Published:** 2025-04-05

**Authors:** Tae-woo Kwon, Guangcheng Wu, Sheng-Nan Lei, J. Fraser Stoddart

**Affiliations:** † Department of Chemistry, The University of Hong Kong, Kowloon 999077, Hong Kong SAR, China; ‡ Department of Chemistry, Northwestern University, 2145 Sheridan Road, Evanston, Illinois 60208, United States; § Center for Regenerative Nanomedicine, Northwestern University, Chicago, Illinois 60611, United States; ∥ Stoddart Institute of Molecular Science, Department of Chemistry, Zhejiang University, Hangzhou 310027, China; ⊥ ZJU-Hangzhou Global Scientific and Technological Innovation Center, Hangzhou 311215, China; # School of Chemistry, University of New South Wales, Sydney, NSW 2052, Australia

## Abstract

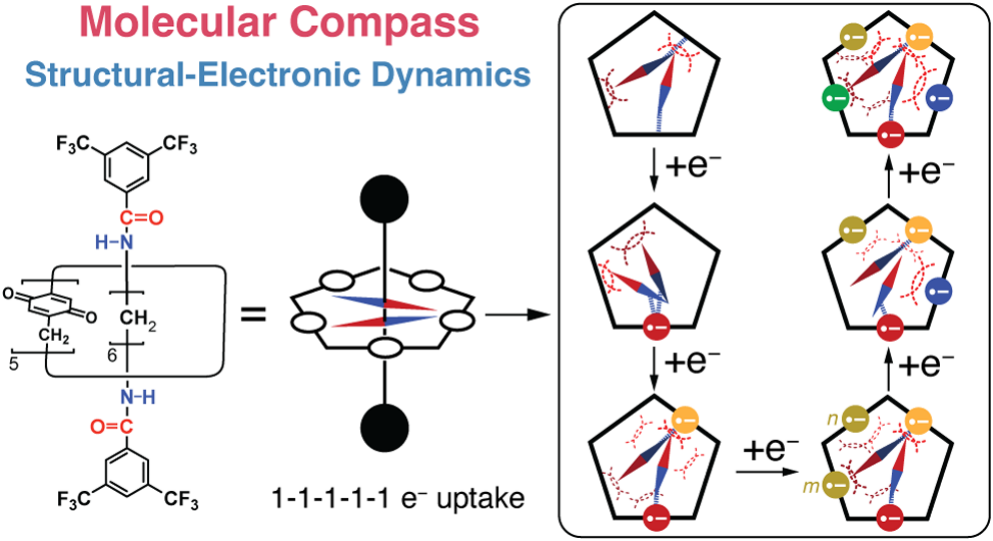

Just as a pointer, which moves freely and points to the magnetic North in a compass, affords us with a device for tracking direction on a global scale, a dipole moment in a molecule is capable of aligning itself in a compass-like manner in response to an electric field at the molecular level. Here, we demonstrate that dipole moment pointers, based on amide and ester groups in the dumbbell components of [2]rotaxanes, are susceptible to changes in pole–dipole and dipole–dipole interactions within a redox-active pillar[5]quinone ring component when subjected to redox control. Distinct from free pillar[5]quinone, these molecular compasses exhibit a 1–1–1–1–1 electron-uptake pattern during the first-electron transfers. Density functional theory (DFT) calculations reveal that, upon the reduction of quinoid units in the amide-based molecular compass, the positive end of the dipole moment pointer in the dumbbell component becomes oriented toward the reduced anionic quinone in the ring component, courtesy of hydrogen bonding. The negative end of the dipole moment pointer in the dumbbell component lowers the reduction potentials of the other four quinoid units as a result of electrostatic repulsions, which explain its 1–1–1–1–1 electron-uptake pattern. Our findings highlight how electronic communication between the dipole moment pointers and the quinoid units in the ring component enables the [2]rotaxane to act as a molecular compass, precisely reorienting its dipole moments in response to redox changes.

## Introduction

A compass is a simple but powerful device for determining direction at a global level. One of the most important parts of a compass is a magnetized pointer that can rotate freely and sense the Earth’s magnetic field by pointing in a northerly and southerly direction. The mechanical operation and function of macroscopic compasses can be emulated at the molecular level by exploiting the dynamics of molecules that transfers force, motion, and energy from one molecule to another. Garcia-Garibay^1^ has designed a freely rotating *p-*phenylene rotor by taking advantage of barrierless alkyne–aryl bond rotations.^2^ Two bulky groups, which act as stators connected to the central rotor, provide steric shielding to facilitate rotation in the crystalline state. If polar groups are incorporated into the *p-*phenylene rotor, their dipoles can respond^1a^ collectively to electric, magnetic, or photonic stimuli. More recently, Chen and Wei^3^ have developed a molecular compass to trace van der Waals interactions within a zeolite. *p-*Xylene, a guest molecule that fits inside the channels in ZSM-5 zeolite frameworks, serves as a rotating pointer and adopts the direction of the lowest interaction energy upon a change in the channel geometry.

**Figure 1:**
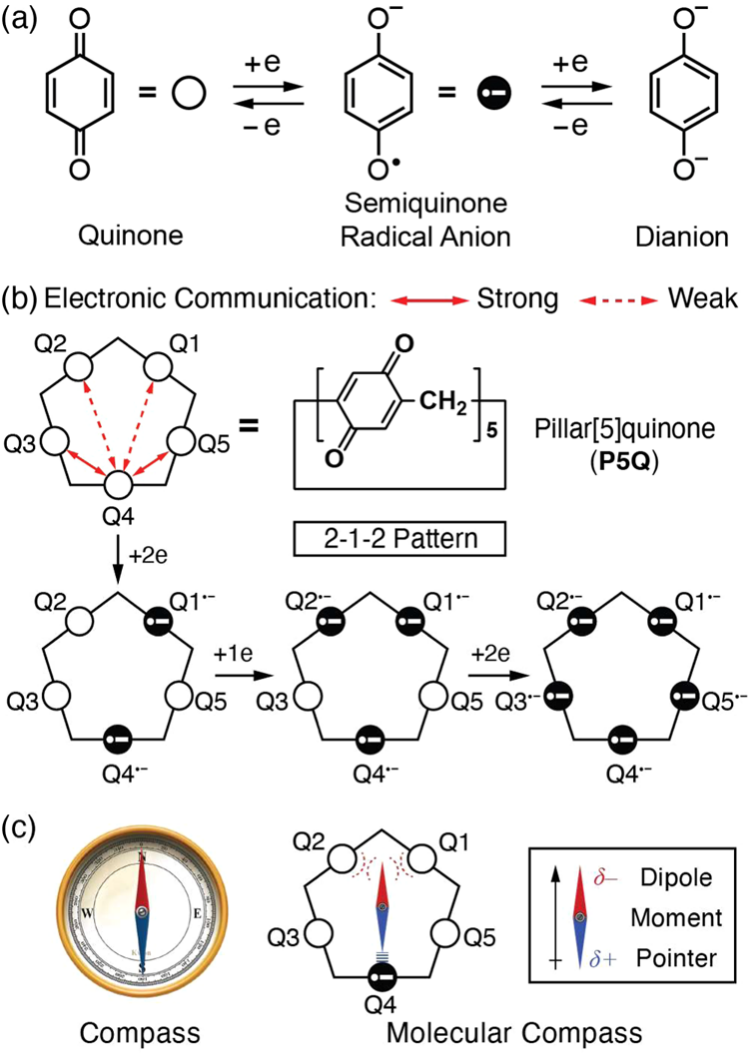
(a) Structural changes of *p*-quinone upon redox reactions in aprotic solvents. *p*-Quinone readily undergoes a one-electron reduction. Note that the reduced quinones possess negative charges on their oxygen atoms. (b) The five quinone redox sites of pillar[5]quinone affect its reduction as a result of electronic communication. How close the quinoid units are to each other determines the strength of their electronic communication. On the one hand, quinones that are distant from each other, such as Q4/Q1 and Q4/Q2, have low electronic coupling, which means they are reduced simultaneously. On the other hand, quinones that are adjacent to each other, such as Q4/Q3 and Q4/Q5, have high electronic coupling, resulting in separate reduction waves with very different potentials. When this principle is applied to the pentagonal arrangement of quinoid units, reductions occur in the sequence of a 2–1–2 electron uptake. (c) The molecular compass operates in a manner similar to a macroscopic compass. It has a dipole moment that acts like a pointer, and its positive end is attracted to the reduced anionic quinones. The negative end of the pointer is oriented away from the reduced quinones. The dipole moment pointer can transmit electronic information from site Q4^•–^ to the distant sites Q1 and Q2 by influencing their electronic states as a result of electrostatic repulsions.

Building upon prior research^1−3^ that demonstrates the directional properties of the compass pointer, we proposed that directionality could be harnessed effectively to convey information regarding the reduction states within a molecule. In this context, pillar[5]quinone attracted our attention because of the redox behavior of its monomer, *p-*benzoquinone. It undergoes^4,5^ (Figure 1a) two successive one-electron transfers in aprotic solvents, resulting in the formation of a radical anion, followed by the formation of a dianion on the reduction of the quinoid unit. Additionally, in pillar[5]quinone, two adjacent quinoid units—e.g., quinones Q4/Q3 and Q4/Q5 in Figure 1b—affect^4,6,7^ the electronic states via through-space electron delocalization and the electrostatic repulsions between negatively charged reduced quinones—also known as semiquinones. This strong electronic coupling makes the reduction of the second quinoid unit more difficult to achieve after the first one has been reduced. In contrast, two distant quinones—e.g., the quinones Q4/Q1 and Q4/Q2 in Figure 1b—that are not connected directly show weak electronic coupling and have similar reduction potentials.^4,6,7^ In general, achieving long-range electronic communication is challenging because electronic-matrix coupling diminishes^8^ as the distance between two redox sites increases. As a result of the nature of these electronic communications between the five quinoid units in pillar[5]quinone, the electron uptake follows^4^ a 2–1–2 sequence as depicted in Figure 1b, i.e., two distant quinones—e.g., Q1 and Q4—are first of all reduced together because of their weak electronic communication. Subsequently, either quinone Q2 or Q3 is reduced preferentially with respect to quinone Q5 because of weaker electrostatic repulsions from the anionic semiquinones Q1^•–^ and Q4^•–^. After quinone Q2 is reduced (Figure 1b), the equivalent quinones Q3 and Q5 are the last to be reduced together.

**Scheme 1:**
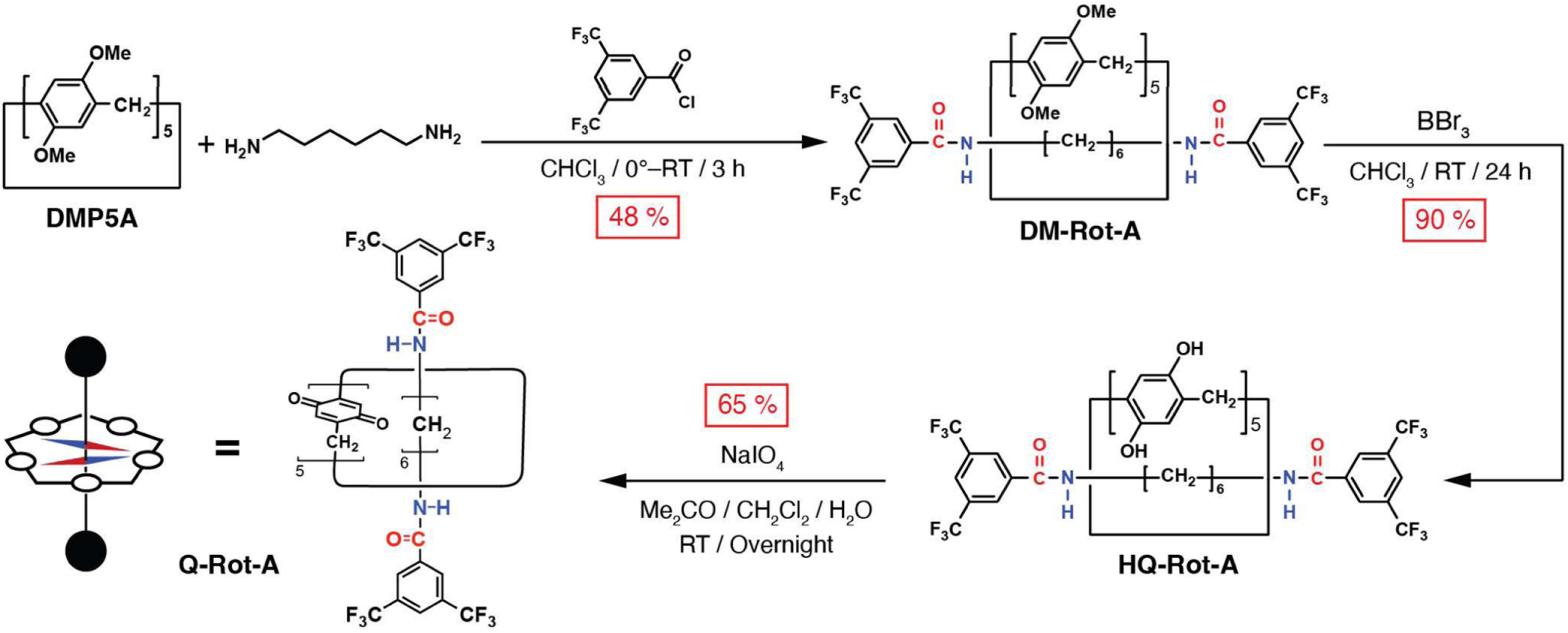
Design and Synthesis of DM-Rot-A, HQ-Rot-A, and Q-Rot-A **DM**, **HQ**, **Q**, **Rot**, and **A** are abbreviations for dimethoxy, hydroquinone, quinone, rotaxane, and amide, respectively.

We envisaged that, if a dipole moment as a pointer is placed in the cavity of pillar[5]quinone just like a compass, the directionality of the dipole moment pointer will reflect (Figure 1c) the reduction potentials of two distant quinones, e.g., Q4/Q1 and Q4/Q2. On the one hand, upon the reduction (Figure 1c) of Q4 to an anionic semiquinone (Q4^•–^), the positive end of the dipole moment points to the anionic semiquinone Q4^•–^ on account of Coulombic attractions. These attractive forces increase the reduction potential of quinone Q4. On the other hand, the negative end of the dipole moment pointer will become directed (Figure 1c) toward quinones Q1 and Q2 on the opposite side from semiquinone Q4^•–^. The HOMO/LUMO levels of the quinones Q1 and Q2 are elevated by the electrostatic repulsions with the pointer, leading to decreases in their reduction potentials. In this manner, the reduced state of quinoid units on one side can be sensed by quinones from afar, e.g., Q4 ↔ Q1/Q2 in Figure 1c. With these considerations in mind, we have designed and synthesized (Schemes 1 and S6) [2]rotaxane-based molecular compasses, **Q-Rot-A** and **Q-Rot-E**, incorporating a pillar[5]quinone as the ring component and either amide bonds (for **Q-Rot-A**) or ester bonds (for **Q-Rot-E**) as part of the dumbbell component. The dipole moments of the amide and ester groups act like pointers and rotate (Scheme 1) around the axis of the dumbbell. Upon the reduction of a quinoid unit, the dipole moment pointers become oriented toward the reduced anionic quinone in the ring component, courtesy of hydrogen-bonding (for **Q-Rot-A**) and electrostatic interactions (for both **Q-Rot-A** and **Q-Rot-E**). This directionality enables remote quinones to communicate with each other regarding their reduction states, leading to a distinct 1–1–1–1–1 pattern of electron uptake.

## Results and Discussion

### Design and Synthesis of Molecular Compasses

In order to place rotatable dipole moment pointers in the center of pillar[5]quinone, we designed a **Q-Rot-A** in Schemes 1 and S1–S3 and **Q-Rot-E** in Schemes S4–S6. Their amide and ester groups in a dumbbell component, which are mechanically interlocked with the pillar[5]quinone by the presence of two bulky 3,5-bis(trifluoromethyl)phenyl stoppers, can rotate freely around the axis of the dumbbell. The amide groups have a large dipole moment^9^ on account of their coplanarity and the high electronegativity of the nitrogen and oxygen atoms. These factors allow the N–H and C=O components of the amide groups to form hydrogen bonds and electrostatic repulsions, respectively, with anionic semiquinones. In contrast, the ester groups in **Q-Rot-E** possess a weaker dipole moment and do not form hydrogen bonds with the quinoid units in the ring component.

A challenge in synthesizing pillar[5]quinone-based rotaxanes is the high reactivity of the quinone unit (a Michael acceptor) with nucleophilic guests. For this reason, the direct use of pillar[5]quinone as a host has been limited when it comes to its rotaxane synthesis. We have adopted another route (Scheme 1) for the synthesis of **Q-Rot-A** using a permethylated pillar[5]arene-based rotaxane **DM-Rot-A**, which was synthesized employing a previously reported procedure^10^ with modifications, resulting in a yield of 48%. In an effort to synthesize **Q-Rot-A**, we explored the oxidative demethylation of the 1,4-dimethoxybenzene units in **DM-Rot-A** using Ce(NH_4_)_2_(NO_3_)_6_. The rotaxane geometry, however, was destroyed, an observation that can be attributed to side reactions involving radical cationic intermediates^11^ of the 1,4-dimethoxybenzene units during oxidation. As an alternative synthetic route that avoids the formation of these radical cationic intermediates, the perhydroxylated pillar[5]arene-based rotaxane (**HQ-Rot-A**) was prepared (Scheme 1) from **DM-Rot-A** by demethylation with BBr_3_ in a 90% yield. The 1,4-dihydroxybenzene units in **HQ-Rot-A** were oxidized^12^ to quinones using NaIO_4_, affording **Q-Rot-A** in a 65% yield. **Q-Rot-E** was synthesized using similar synthetic routes. Fortunately, we identified a reaction condition under which the ester bond remains intact in the presence of NaHCO_3_ during the demethylation of **DM-Rot-E** to **HQ-Rot-E** using the Lewis acid BBr_3_. Further details of the syntheses and characterizations are described in the Supporting Information (see Schemes S1–S6 and Figures S1–S7).

### Structural Studies

**Figure 2:**
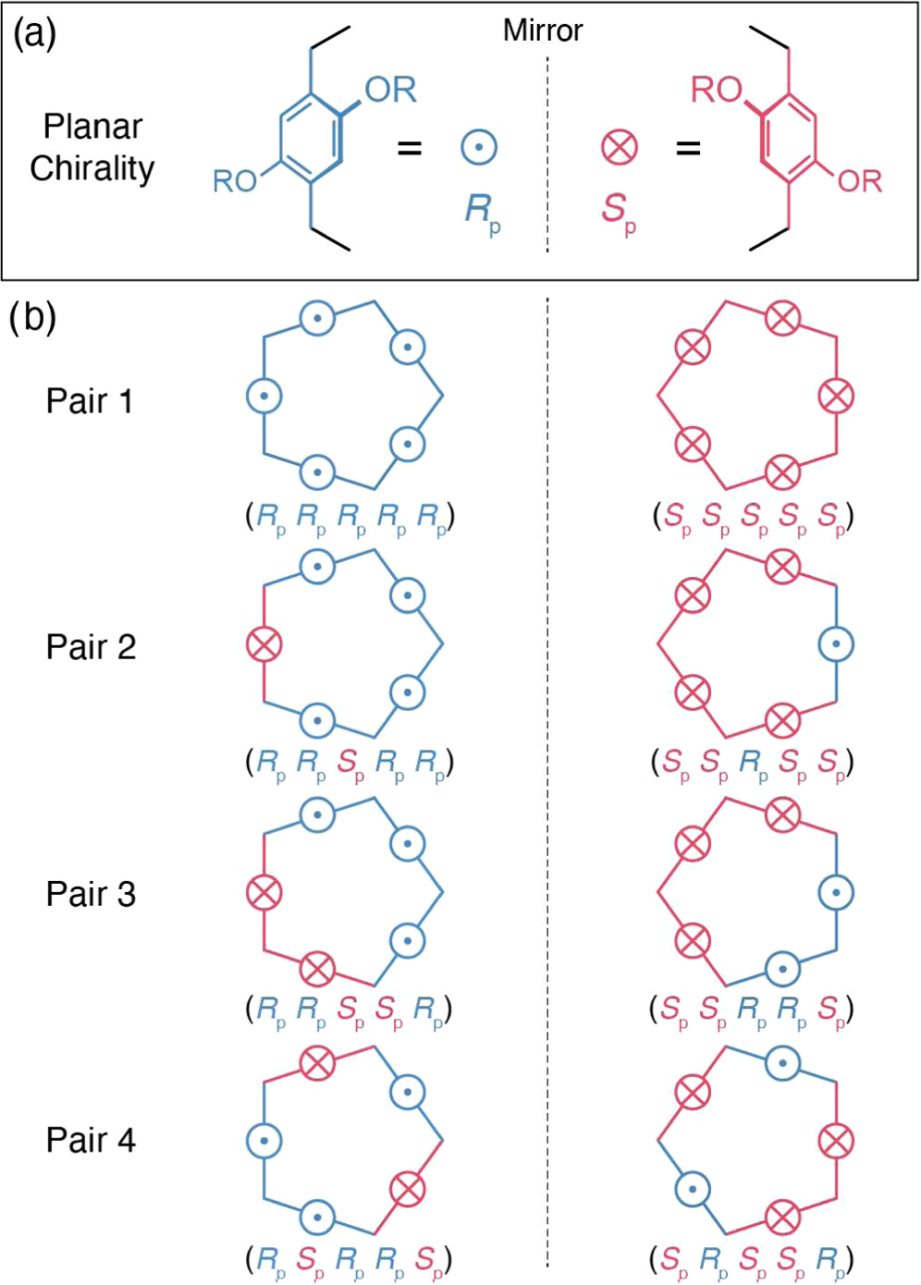
(a) Planar chirality (*S*_p_ and *R*_p_) of the phenylene units in pillar[5]arene, which arises because of the orientation of its 2,5-substituents. *S*_p_ and *R*_p_ are nonsuperimposable mirror images of each other. (b) The eight possible atropisomers of pillar[5]arene emerge when the rotations of phenylene unit are restricted. Each atropisomer of pillar[5]arene has a nonsuperimposable mirror image, allowing them to be classified into four enantiomeric pairs.

Pillar[5]arenes, initially introduced into the chemical literature by Ogoshi et al.^13a^ in 2008, have undergone extensive investigation^13^ to elucidate their structural, stereochemical, and host–guest binding properties. Because the orientation of substituents of phenylene units at the 2- and 5-positions breaks (Figure 2a) molecular symmetry, pillar[5]arenes exist^14,15^ as numerous planar chiral stereoisomers when the oxygen-through-the-annulus rotation of the phenylene units is restricted. Each phenylene unit can be designated to be either *R*_p_ or *S*_p_. The combination (Figure 2b) of these five planes of chirality in pillar[5]arenes generates^14^ theoretically eight atropisomers, i.e., four pairs of enantiomers.^13b,13c^

The phenylene units in permethylated pillar[5]arene (**DMP5A**) have been reported^13d^ to undergo oxygen-through-the-annulus rotation even at low temperatures (e.g., −90 °C) on account of weak interactions between the methoxy groups. Although this observation suggests that **DMP5A** can, in principle, adopt all of the eight stereoisomers, only the enantiomeric pair 1 (Figure 2b) was observed on the ^1^H NMR time scale, implying the fast transition^13b,13c^ between (*R*_p_*R*_p_*R*_p_*R*_p_*R*_p_) and (*S*_p_*S*_p_*S*_p_*S*_p_*S*_p_) because of the methoxy–methoxy steric interactions between adjacent phenylene units.

A ring component, i.e., DMP5A, of **DM-Rot-A** inherits (Scheme 1) the conformations of the free DMP5A upon stoppering. As with proton signals for the free **DMP5A**, signals for the phenylene units and methylene bridges of DMP5A in **DM-Rot-A** were observed (Figure S1) as well-defined singlets at 6.92 and 3.76 ppm, respectively. These singlet proton resonances indicate that all five phenylene units of the ring component with the D_5_ local symmetry of **DM-Rot-A** have the same planar chirality, i.e., **DM-Rot-A** has (*R*_p_*R*_p_*R*_p_*R*_p_*R*_p_) or (*S*_p_*S*_p_*S*_p_*S*_p_*S*_p_) co-configuration (enantiomeric pair 1 in Figure 2b) that is a racemic modification as confirmed (Figure S13) by the optical inactivity in its circular dichroism (CD) spectrum. Inversion between (*R*_p_*R*_p_*R*_p_*R*_p_*R*_p_)- and (*S*_p_*S*_p_*S*_p_*S*_p_*S*_p_)-**DM-Rot-A** is not possible on account of the unavailability of oxygen-through-the-annulus rotation of its ring component by the dumbbell component.

**Figure 3:**
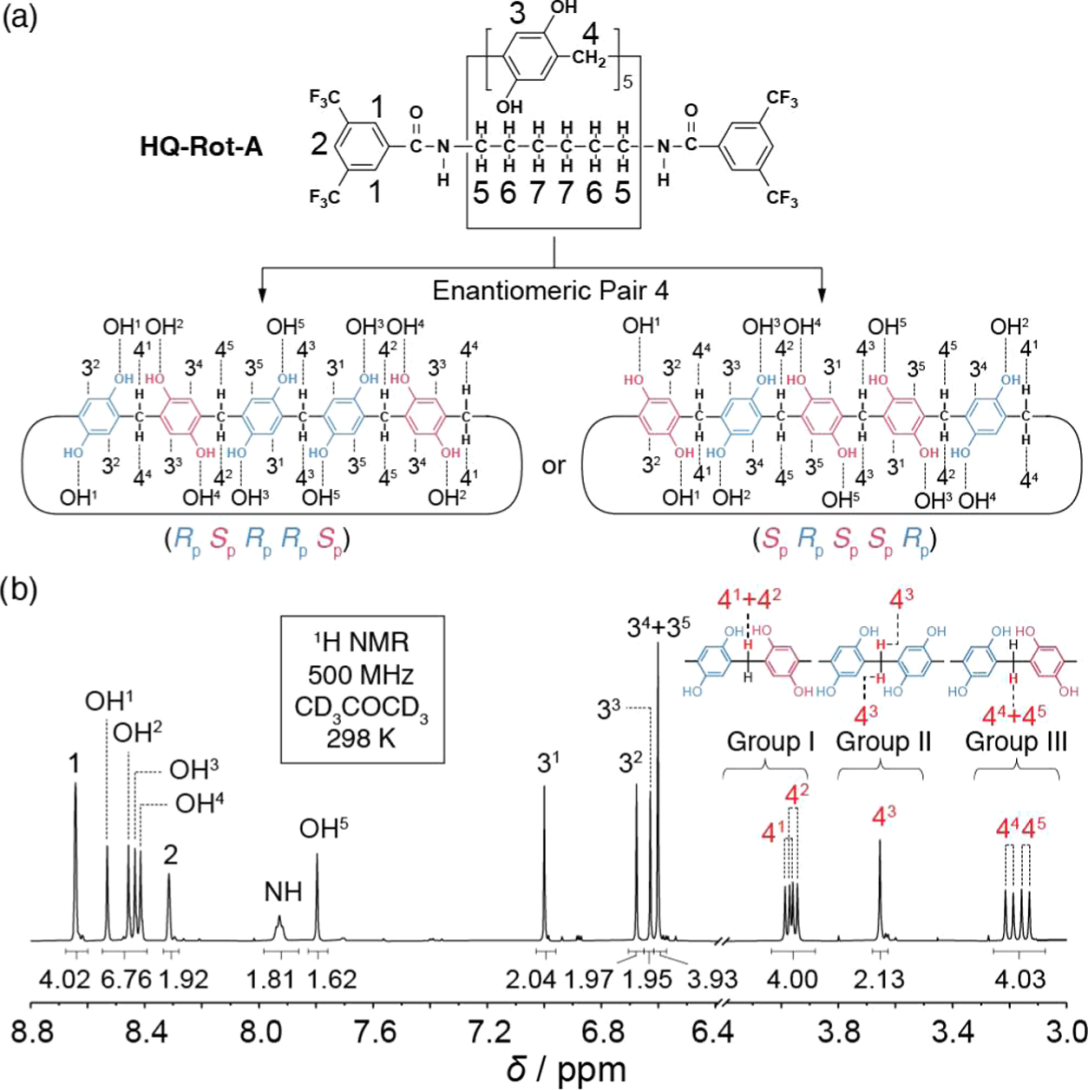
Structural formula and ^1^H NMR spectrum of HQ-Rot-A. (a) The structural formula of HQ-Rot-A showing its enantiomeric pairs (*R*_p_*S*_p_*R*_p_*R*_p_*S*_p_)- and (*S*_p_*R*_p_*S*_p_*S*_p_*R*_p_)-HQ-Rot-A. The ring component configuration for each enantiomer is depicted below the structure with the protons 3^1–5^, 4^1–5^, and OH^1–5^ labeled. (b) Partial ^1^H NMR spectrum (500 MHz, CD_3_COCD_3_, 298 K) of HQ-Rot-A. There are three different types of protons 4, designated as groups I, II, and III. The integral ratio of the group I: II: III = 4:2:4 matches the structure of the enantiomeric pair 4. The hydrogen bonds with nearby OH groups result in the downfield shift of the OH^1–4^, proton 3^1^, and 4^1–3^. 2D ^1^H–^1^H NOESY NMR (Figure 4) and COSY NMR (Figure S7) spectroscopies were used to assign protons. Figures S3 and S4 present the full ^1^H NMR and ^13^C NMR spectra for HQ-Rot-A, respectively.

The co-configurations of **HQ-Rot-A** were analyzed (Figures 3 and S3) by recoding ^1^H NMR spectroscopy. As **HQ-Rot-A** was synthesized directly from **DM-Rot-A**, the co-configuration of **HQ-Rot-A** was expected initially to be the same as that of **DM-Rot-A**. The ^1^H NMR spectrum (Figure 3) for **HQ-Rot-A**, however, shows five resonances for the phenylene units (peaks 3^1^–3^5^), methylene bridges (peaks 4^1^–4^5^), and hydroxyl groups (peaks OH^1^–OH^5^). These multiple resonances indicate that the planes of chirality associated with the **HQ-Rot-A**’s ring component are a mixture of *R*_p_ and *S*_p_ planar chiralities. In order to assign them, we ascribe the proton resonances (4^1^–4^5^) for methylene bridges into three groups (Figure 3b) based on their local chemical environments: (i) group I: protons encompassing by two neighboring hydroxyl groups, (ii) group II: protons in close proximity to only one hydroxyl group, and (iii) group III: protons that are distant from two hydroxyl groups. The proton resonances for group I (4.1–3.9 ppm, protons 4^1^ and 4^2^) and group II (3.7–3.6 ppm, proton 4^3^) are shifted downfield, compared to those for group III (3.3–3.1 ppm, protons 4^4^ and 4^5^), because of the deshielding that occurs as a result of their hydrogen bonding to OH groups. Among the possible eight stereoisomers (Figure 2b), only enantiomeric pair 4 has the ratio of group I: group II: group III = 4:2:4, an observation that is consistent (Figure 3b) with the integrated ratio for protons 4^1^–4^5^ in **HQ-Rot-A**. Moreover, in the case of the enantiomeric pair 4, the protons in the methylene bridging units in groups I and III are diastereotopic and are doublets with geminal coupling constants, while the protons in the methylene bridging units in group II are homotopic and resonate as a singlet. Similar to the trend observed in the shift of peaks 4^1–5^, the downfield shifts of peak 3^1^ and OH^1^–OH^4^ compared to those in 3^2^–3^5^ and OH^5^, respectively, can also be interpreted in terms of deshielding as a result of hydrogen bonding with hydroxyl groups. The CD inactivity (Figure S13b) of **HQ-Rot-A** suggests that (*R*_p_*S*_p_*R*_p_*R*_p_*S*_p_)- and (*S*_p_*R*_p_*S*_p_*S*_p_*R*_p_)-**HQ-Rot-A** are a mixture of enantiomers.

**Figure 4:**
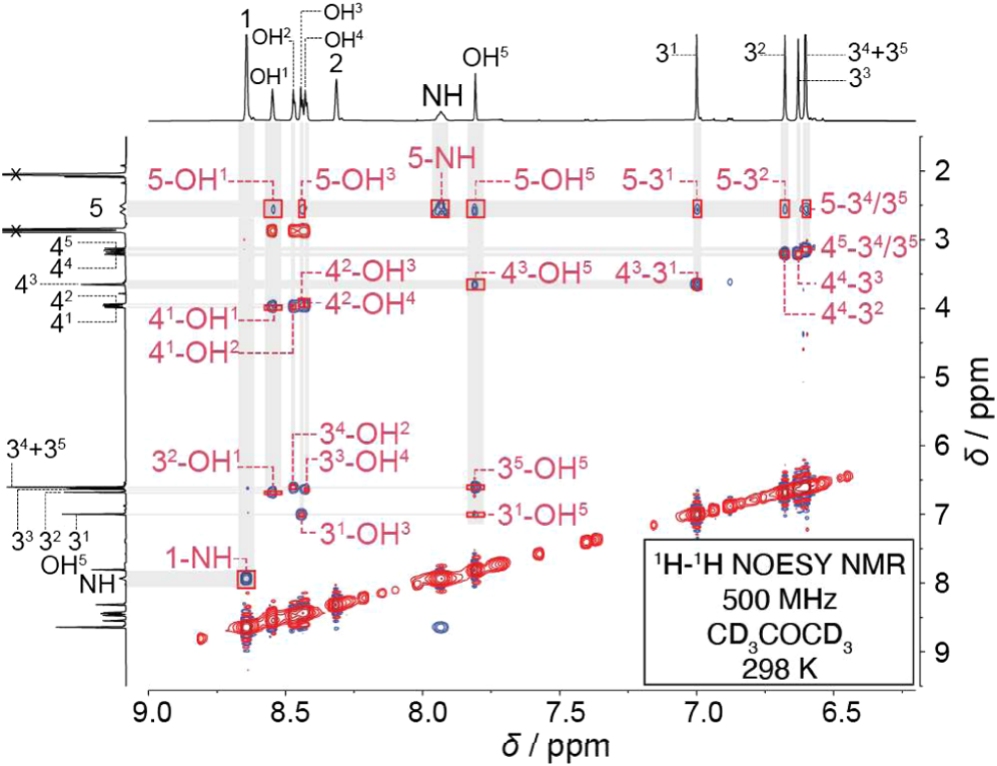
2D ^1^H–^1^H NOESY spectrum (500 MHz, CD_3_COCD_3_, 298 K, mixing time = 0.3 s) of HQ-Rot-A. The accurate assignment of protons 3^1–5^, 4^1–5^, and OH^1–5^ is enabled by the NOE signals between them. This assignment verifies that HQ-Rot-A is an enantiomeric pair 4.

In order to assign the other peaks (3^1^–3^5^ and OH^1^–OH^5^) of **HQ-Rot-A**’s ring component in its ^1^H NMR spectrum (Figures 3b and S3), 2D ^1^H–^1^H NOESY spectroscopy (Figure 4) was employed. The protons 4^1^ and 4^2^ (group I in Figure 3b) in methylene bridging units exhibited NOE correlation signals with two pairs of protons on hydroxyl groups, OH^1^/OH^2^ and OH^3^/OH^4^, respectively (refer to Figure 3a for its structural formula). This observation indicates (Figure 3a) that the hydroxyl groups on the two neighboring phenylene units are oriented toward protons 4^1^ and 4^2^. This structural arrangement is supported by the absence of NOE signals between protons 4^1^ and 4^2^ and any of the phenylene protons 3. Proton 4^3^ exhibits (Figure 4) a strong NOE signal with only one (OH^5^) of the hydroxyl groups, indicating that it belongs to group II (Figure 3b). Furthermore, protons 4^4^ and 4^5^ exhibit no cross peaks with OH groups, implying that these protons are positioned at a distance from any OH groups and confirming their assignment to group III. In place of OH groups, protons 3^2^/3^3^ and 3^4^/3^5^ are positioned close to protons 4^4^ and 4^5^, respectively, as confirmed (Figure 4) by their NOE signals between 4^4^ and 3^2^/3^3^ and between 4^5^ and 3^4^/3^5^. The NOE correlations observed between protons 3^1–5^ and protons on the OH^1–5^ also support the configuration arrangements of the enantiomeric pair 4. The phenylene proton 3^1^ exhibits cross peaks with two of the protons (OH^3^ and OH^5^) on the OH groups, whereas protons 3^2^–3^5^ each have cross peaks with one of the protons (OH^1^, OH^2^, OH^4^, and OH^5^, respectively) on the OH groups. Furthermore, the ^1^H–^1^H COSY NMR spectrum (Figure S7) of **HQ-Rot-A** reveals the geminal nature of the diastereotopic methylene protons 4^1^/4^4^ and 4^2^/4^5^ and the homotopicity of methylene protons 4^3^.

**Figure 5:**
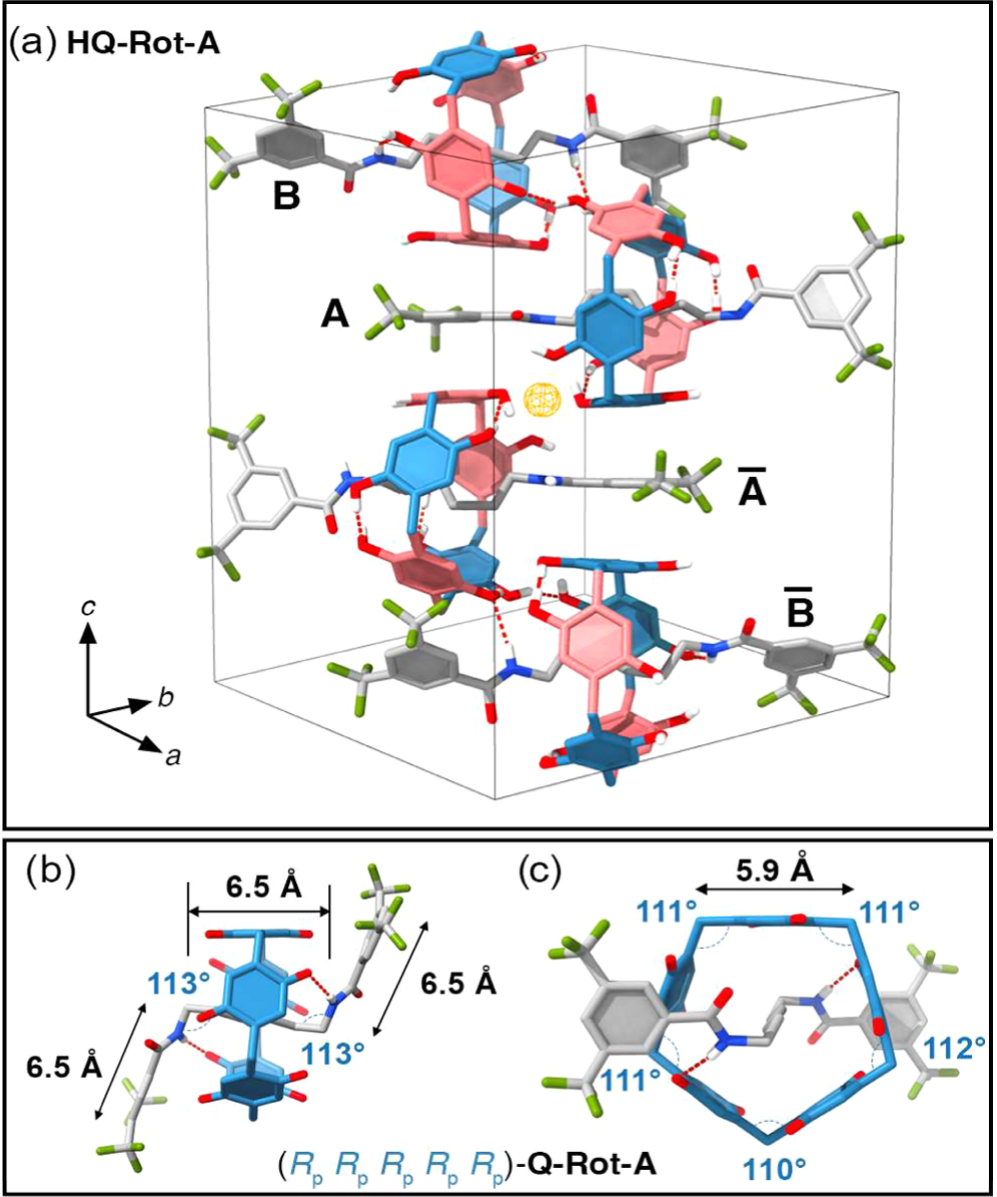
Tubular representations of the solid-state structure of HQ-Rot-A and Q-Rot-A. (a) The unit cell of HQ-Rot-A, with space group *P*1̅, contains four HQ-Rot-A molecules. A and B are mirror images of A̅ and B̅ in the solid state, respectively, with a center of inversion represented by the orange sphere at the center of the unit cell. The co-conformers A and B̅ exhibit a (*R*_p_*S*_p_*R*_p_*R*_p_*S*_p_) co-configuration, whereas the co-conformers A̅ and B display a (*S*_p_*R*_p_*S*_p_*S*_p_*R*_p_) co-configuration. (b) Side-on and (c) front views of (*R*_p_*R*_p_*R*_p_*R*_p_*R*_p_)-Q-Rot-A reveal that N–H bonds on the dumbbell form intercomponent hydrogen bonds with the C=O groups of the pillar[5]quinone, causing the axle component to bend at an angle of 113°. Hydrogen atoms are omitted for the sake of clarity except for those in OH and NH groups for HQ-Rot-A and NH groups for Q-Rot-A. Red dotted lines indicate hydrogen bonds. Blue and pink phenylene units of ring components denote *R*_p_ and *S*_p_ planar chiralities, respectively.

The solid-state structure of **HQ-Rot-A** was determined by single-crystal X-ray crystallography in order to reconfirm the co-configurations of **HQ-Rot-A**. Single crystals of **HQ-Rot-A** were obtained by the slow diffusion of CH_2_Cl_2_ into a solution of **HQ-Rot-A** in MeOH. The solid-state (super)structure of **HQ-Rot-A** reveals that the unit cell (space group *P*1̅) contains (Figure 5a) four molecules including two enantiomeric pairs (A and A̅, B and B̅) that are related by a center of inversion in the unit cell. Interestingly, B and B̅ are co-conformers with A̅ and A, respectively, wherein their ring components are shifted, enabling intermolecular hydrogen bonding between the NH groups on their dumbbell components of B and B̅ and the hydroxyl groups on the ring components of A and A̅. Thus, consistent with the co-configuration of **HQ-Rot-A** determined through the NMR spectroscopy, the crystal structure demonstrates that co-conformers A and B̅ possess the same (*R*_p_*S*_p_*R*_p_*R*_p_*S*_p_) co-configuration, while co-conformers A̅ and B exhibit the (*S*_p_*R*_p_*S*_p_*S*_p_*R*_p_) co-configuration.

The alteration in planar chirality of the ring component during the conversion of **DM-Rot-A** to **HQ-Rot-A** suggests that the perhydroxylated phenylene units in the ring components of **HQ-Rot-A** undergo oxygen-through-the-annulus rotations, a behavior not observed in **DM-Rot-A**. In the previous literature,^13e^ it was reported that a perhydroxylated pillar[5]arene exhibits slow oxygen-through-the-annulus rotation in the presence of a bound linear aliphatic chain. Based on this observation, it is not unreasonable to infer that the perhydroxylated phenylene groups of a **HQ-Rot-A** can undergo rotation, enabling hydrogen bonding between them and resulting in a thermodynamically stable configuration^13f^ of mixed *R*_p_ and *S*_p_. The stability of this co-configuration is supported by the fact that proton resonances in **HQ-Rot-A** retain the same chemical shifts, even at elevated temperatures in different deuterated solvents—specifically, at 45 °C in CD_3_COCD_3_, 60 °C in CD_3_CN, and 140 °C in CD_3_SOCD_3_ (Figures S8–S10).

The ^1^H NMR spectrum (Figure S5) of **Q-Rot-A** shows that the observed conformations^16^ of its ring component differ from configurations for **HQ-Rot-A**. Singlet proton peaks for phenylene (proton 3 at 6.77 ppm) and methylene bridges (proton 5 at 3.49 ppm) indicate that all phenylene units on its ring components adopt symmetrical conformations,^16^ specifically (*R*_p_*R*_p_*R*_p_*R*_p_*R*_p_) or (*S*_p_*S*_p_*S*_p_*S*_p_*S*_p_). The quinoid unit of **Q-Rot-A** is most likely rotatable on account of its C=O bond length (1.22 Å) being shorter than the C–OH bond length (1.39 Å) of **HQ-Rot-A**. The analysis of the NMR spectroscopic data is in good agreement with the single-crystal X-ray structure (Figure 5b,c) of **Q-Rot-A**. While Figure 5b,c selectively displays the structural information for (*R*_p_*R*_p_*R*_p_*R*_p_*R*_p_)-**Q-Rot-A**, the disorder in its solid-state structure indicates that (*R*_p_*R*_p_*R*_p_*R*_p_*R*_p_) and (*S*_p_*S*_p_*S*_p_*S*_p_*S*_p_) conformations^16^ of **Q-Rot-A** are distributed within a single crystal in a 1:1 ratio. Both ends of the dumbbell component are bent (Figure 5c) at 113° as a result of the intercomponent hydrogen bonds between NH groups of the dumbbell and carbonyl groups of the ring components. The lengths of sides of the ring component in **Q-Rot-A**, which are 5.9 Å, are slightly larger than those (5.8–5.9 Å) in **HQ-Rot-A**. The contraction for **HQ-Rot-A** can be explained by strong intramolecular hydrogen bonds between perhydroxylated phenylene groups in **HQ-Rot-A**. The sides of the ring components in **Q-Rot-A** are convex (Figure 5c) with the inside angles larger (110, 111, and 112°) than those (108°) of a regular pentagon.

### Electrochemistry

**Figure 6:**
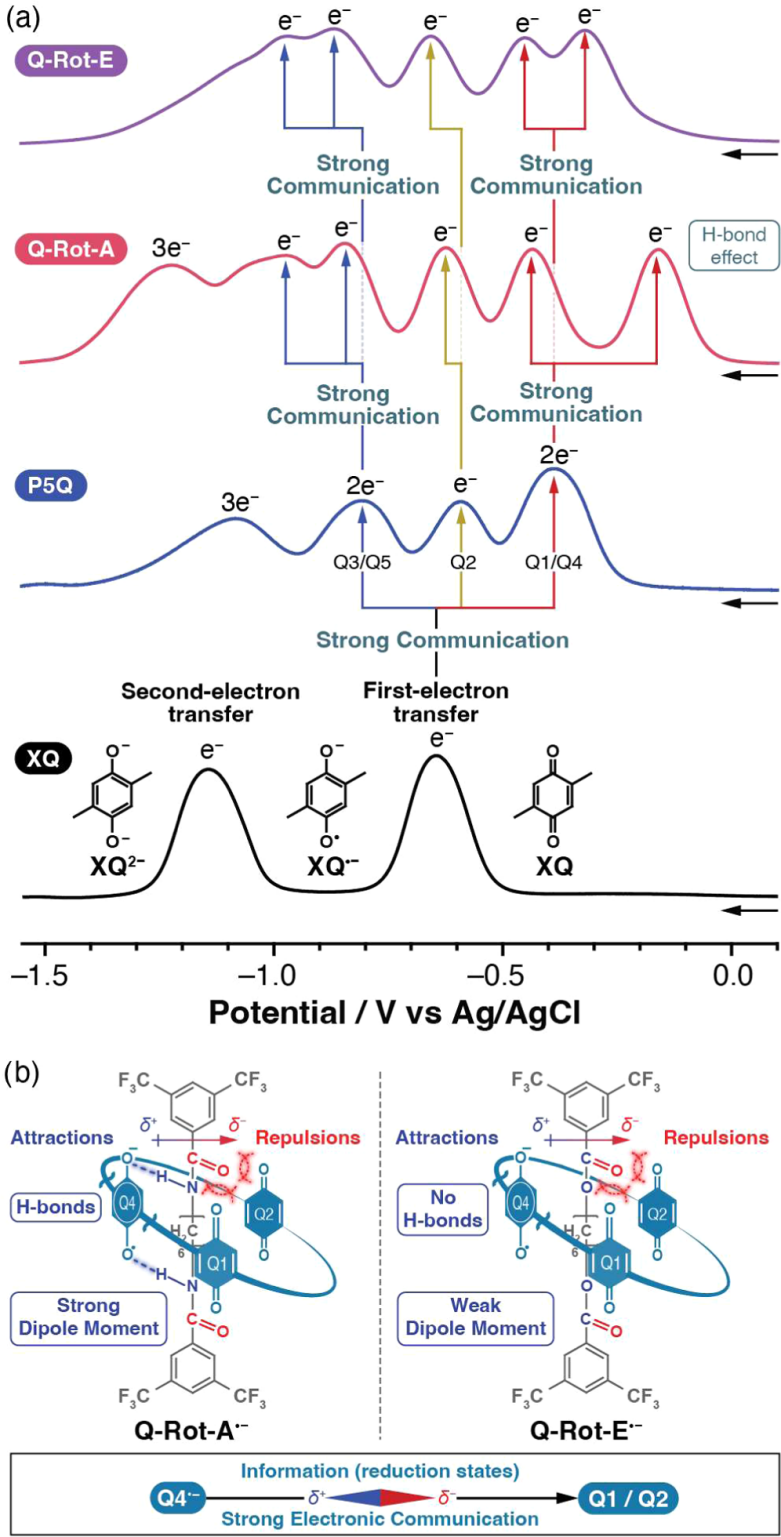
(a) Square-wave voltammograms (SWVs) for the reduction of XQ, P5Q, Q-Rot-A, and Q-Rot-E (1 mM in CH_2_Cl_2_ including 0.1 M Bu_4_NPF_6_ as the supporting electrolyte), showing the reduction processes. The observed splitting of reduction waves indicates strong electronic communication between quinoid units. Q1–Q5 represents the specific quinoid units illustrated in Figure 1b. (b) Structural formulas of Q-Rot-A^•–^ and Q-Rot-E^•–^ illustrating the alignment of dipole moments. These alignments, induced by the reduction of Q4, direct the C=O bonds toward remote quinoid units (Q1 and Q2), thereby enhancing electronic communication. For the sake of clarity, the quinoid units Q3 and Q5 are omitted.

Square-wave voltammograms (SWVs) for the reduction of *p*-xyloquinone (**XQ**), **P5Q**, **Q-Rot-A**, and **Q-Rot-E** were recorded (Figure 6) over a potential range of 0.10 and −1.55 V vs Ag/AgCl in order to substantiate the operation of the molecular compasses (**Q-Rot-A** and **Q-Rot-E**) and its influence on the electronic communication between the quinones. All potentials hereafter are reported with respect to Ag/AgCl. Detailed experimental conditions and parameters are described in the Supporting Information. **XQ**, whose structure is the same as the monomer of **P5Q**, exhibited (Figure 6a) two consecutive one-electron reductions at −0.65 and −1.14 V in CH_2_Cl_2_. The first-electron transfer at −0.65 V is related to the conversion of **XQ** to its radical anion (**XQ**^•**–**^), and the second-electron transfer at −1.14 V is involved in the conversion of **XQ**^**•–**^ to its dianion (**XQ**^**2–**^). It is known^4,17^ that the first-electron transfer is suited to the quantitative electrochemical analyses owing to its good reversibility, while the second-reduction wave is less well developed and irreversible in many cases because of its sensitivity toward residual protic species present in an electrochemical cell. For this reason, we have focused on the electronic coupling occurring during the first-electron transfer involving quinoid units in **P5Q**, **Q-Rot-A**, and **Q-Rot-E**. We use the term “first-electron transfer” in this article to denote the reduction process (Q → Q^•–^) wherein quinones (Q) are converted to their semiquinones (Q^•–^). It should be made clear that the first-electron transfer does not refer to the first reduction wave in the first-electron-transfer processes involving the five quinones for **P5Q**, **Q-Rot-A**, and **Q-Rot-E**.

The influence of the pentagonal symmetry of **P5Q** on the first-electron transfer has been investigated by Kaifer et al.^4,7,18^ Consistent with their results of a 2–1–2 electron-uptake pattern, two, one, and two quinoid units in **P5Q** are reduced successively (Figure 6a) at −0.39, −0.59, and −0.81 V, respectively. These reduction waves correspond to the first-electron transfers in each quinoid unit, generating semiquinone radical anions (Q → Q^•–^) in the sequence of Q1/Q4 → Q2 → Q3/Q5, as delineated in Figure 1b. The redox potential splitting reflects the intramolecular electronic coupling between quinoid units. Identical redox sites with weak or no coupling exhibit^18,19^ overlapping voltammetric waves, as observed (Figure 6a) in the first (Q1 and Q4) and third (Q3 and Q5) reduction waves. Conversely, strong electronic coupling increases^20,21^ the separation between redox potentials, resulting in distinct voltammetric waves and the observed separation into three reduction waves for **P5Q**. As a general rule, adjacent quinones communicate^4,6,7,18,22^ strongly by through-space electron delocalization and electrostatic interactions, while remote quinones have weak electronic coupling. It is also important to note that the first- and second-reduction waves of **P5Q** occur (Figure 6a) at higher potentials (−0.39 and −0.59 V, respectively) than that of **XQ** (−0.65 V) due to the electron-withdrawing inductive effect of neighboring quinoid units, while the third wave is shifted to a lower potential by electrostatic repulsions from the three reduced quinoid units (Q1^•–^, Q2^•–^, and Q4^•–^ in Figure 1b).

In an effort to enable electronic communication between remote quinones in **P5Q**—such as Q4 with Q1 and Q2 during the first reduction, and Q3 with Q5 during the third reduction—where inherent electronic coupling is weak, amide groups have been deployed strategically within the cavity of P5Q in the form of a [2]rotaxane (**Q-Rot-A**). This design facilitates specific interactions between remote quinoid units, resulting in five distinct reduction waves during the first-electron transfers.

In order to ascertain the number of electrons associated with each reduction wave for **Q-Rot-A**, we employed normal pulse voltammetry (NPV), a technique that can measure plateau currents during each redox reaction in response to the constantly increasing potential pulsing steps. The plateau current (*i*) can be described by the Cottrell equation^23^
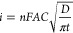
1where *n* is the number of electrons, *F* is Faraday’s constant, *A* is the electrode area, *C* is the concentration, *D* is the diffusion coefficient, and *t* is time. If the same experimental conditions for the electrode area (*A*), concentration (*C*), and time parameter (*t*) are employed in the case of **XQ** and **Q-Rot-A**, this relationship can be rearranged^4,18^ to give
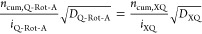
2

It is well known^4^ that **XQ** undergoes two one-electron reactions during its full reduction—that is, *n*_cum,XQ_ = 2. Its NPV shows (Figure S14a) that the plateau current (*i*_XQ_) is 132.6 μA upon full reduction. The diffusion coefficients for **XQ** (*D*_XQ_) and **Q-Rot-A** (*D*_Q-Rot-A_) in the voltammetry cells were determined to be 2.42 × 10^–5^ and 1.17 × 10^–5^ cm^2^ s^–1^, respectively, by the diffusion-ordered ^1^H NMR spectroscopy (DOSY) measurements (Figures S11 and S12) corrected using the Stokes–Einstein equation (see section D of the Supporting Information for details). From these values, the cumulated numbers (*n*_cum,Q-Rot-A_) of electrons were calculated based on eq 2 and are summarized in Table 1. The voltammetric waves 1–5 for **Q-Rot-A** correspond to the first-electron transfer (Q → Q^•–^) with a one-electron stoichiometry (*n*_Q-Rot-A_ = 1). The sixth wave originates from second-electron transfers (Q^•–^ → Q^2–^) with a three-electron stoichiometry (*n*_Q-Rot-A_ ≈ 3).

**Table 1:** Electrochemical Data for Voltammetric Waves of Q-Rot-A

wave^a^	*E*_red_^b^ V vs Ag/AgCl	*i*_Q-Rot-A_^c^ (μA)	*n*_cum,Q-Rot-A_^d^	*n*_Q-Rot-A_^e^
1	–0.16	46.0	1.0	1.0
2	–0.44	94.0	2.0	1.0
3	–0.62	138.2	3.0	1.0
4	–0.84	228.4	5.0	2.0
5	–0.97			
6	–1.22	355.9	7.7	2.8

a Voltammetric waves are numbered from high to low potential in the SWV (Figure 6a) of **Q-Rot-A**. Waves 1–5 correspond to the first-electron transfer (Q → Q^•–^) of each quinone unit, while wave 6 corresponds to the second-electron transfer (Q^•–^ → Q^2–^).

b Obtained from the peak potential values in the SWV (Figure 6a) of **Q-Rot-A**.

c Plateau currents are determined from the NPV of **Q-Rot-A** (Figure S14b).

d Cumulative number of electrons calculated employing eq 2.

e Number of electrons (*n*_Q-Rot-A_) involved in each voltammetric wave.

The voltammetric behavior of **Q-Rot-A** supports the operation of a molecular compass, which features dipole moment pointers (amide bonds in the dumbbell component) that modify the electronic properties of quinoid units in the ring component. In the neutral state of **Q-Rot-A**, the dipole moment pointers exhibit rapid rotation over the quinoid units on the NMR time scale at 298 K, as evidenced by the sharp singlet observed for the quinone protons in its ^1^H NMR spectrum (Figure S5). Concurrently, these dipole moment pointers participate in hydrogen bonding with the quinoid units, as confirmed by solid-state structural analysis (Figure 5b,5c), thereby enhancing the reduction potentials of the quinoid units. As a result, the first reduction of the quinoid units was observed at – 0.16 V (Figure 6a), a value notably higher than the −0.39 V recorded for **P5Q**. The first-electron transfer (Q → Q^•–^) of **Q-Rot-A** generates (Figure 1a) a negative charge on the oxygen atom of the semiquinone unit, strengthening hydrogen bonds with the N–H bonds in the amide groups. Furthermore, the orientation of the N–H bond toward the anionic semiquinone causes the C=O group to point away (Figure 6b) toward remote quinones (Q1/Q2) on the opposite side of P5Q. The partial negative charge of the C=O group impedes the reduction of the nearby quinoid units on account of electrostatic repulsions, consequently decreasing their reduction potentials. As a result of these electrostatic repulsions, the reduction wave (−0.44 V) of the second quinoid unit is separated (Figure 6a) from that (−0.16 V) of the first quinoid unit for **Q-Rot-A**, demonstrating the strong electronic communication between the remote quinoid units (Q1 and Q4). The third reduced quinoid unit in **Q-Rot-A** exhibits a reduction potential of –0.62 V, which is slightly lower than the reduction potential (−0.59 V) observed for **P5Q** as a result of electrostatic repulsions. Remarkably, although the last two quinoid units (Q3 and Q5, Figure 1b) in free **P5Q** possess identical structural environments, their electrostatic environments differ in **Q-Rot-A** due to the orientation of the dipole moment pointer. The quinoid unit farther from the C=O bond is more favorable for the next reduction on account of its weaker electrostatic repulsions with the dipole moment pointer. The reduction potential of this quinoid unit was recorded (Figure 6a) at a lower value of −0.84 V compared to that (−0.81 V) for **P5Q**, suggesting that it is also affected to some degree by the electrostatic repulsions from the C=O group in the dipole moment pointer. The last quinoid unit in **Q-Rot-A** has a much lower reduction potential of −0.97 V by dint of the strong Coulombic repulsions from the C=O group. The voltammetric behavior of **Q-Rot-A** reveals that the directionality of the molecular compass’s pointer serves as an information channel (the inset of Figure 6b) regarding the reduction states of the quinoid units. This compass operation enables a long-range electronic communication between remote quinoid units, where the reduction states of each quinoid unit influence the others, culminating in a sequential electron-uptake pattern of 1–1–1–1–1 (Figure 6a).

A similar 1–1–1–1–1 electron-uptake pattern is observed for **Q-Rot-E**, which features ester groups, with reduction potentials at −0.32, −0.45, −0.66, −0.87, and −0.97 V (Figure 6a). This pattern arises from the attractive and repulsive interactions between the quinoid units and the dipole moments of the ester groups. Unlike **Q-Rot-A**, however, **Q-Rot-E** lacks hydrogen bonding, leading to a smaller shift (Figure 6a) in the first reduction potential compared to **Q-Rot-A**. This difference supports the conclusion that the increase in the first reduction potential of **Q-Rot-A** is associated with hydrogen bonding with the dipole moment pointer.

### Computational Studies

**Figure 7:**
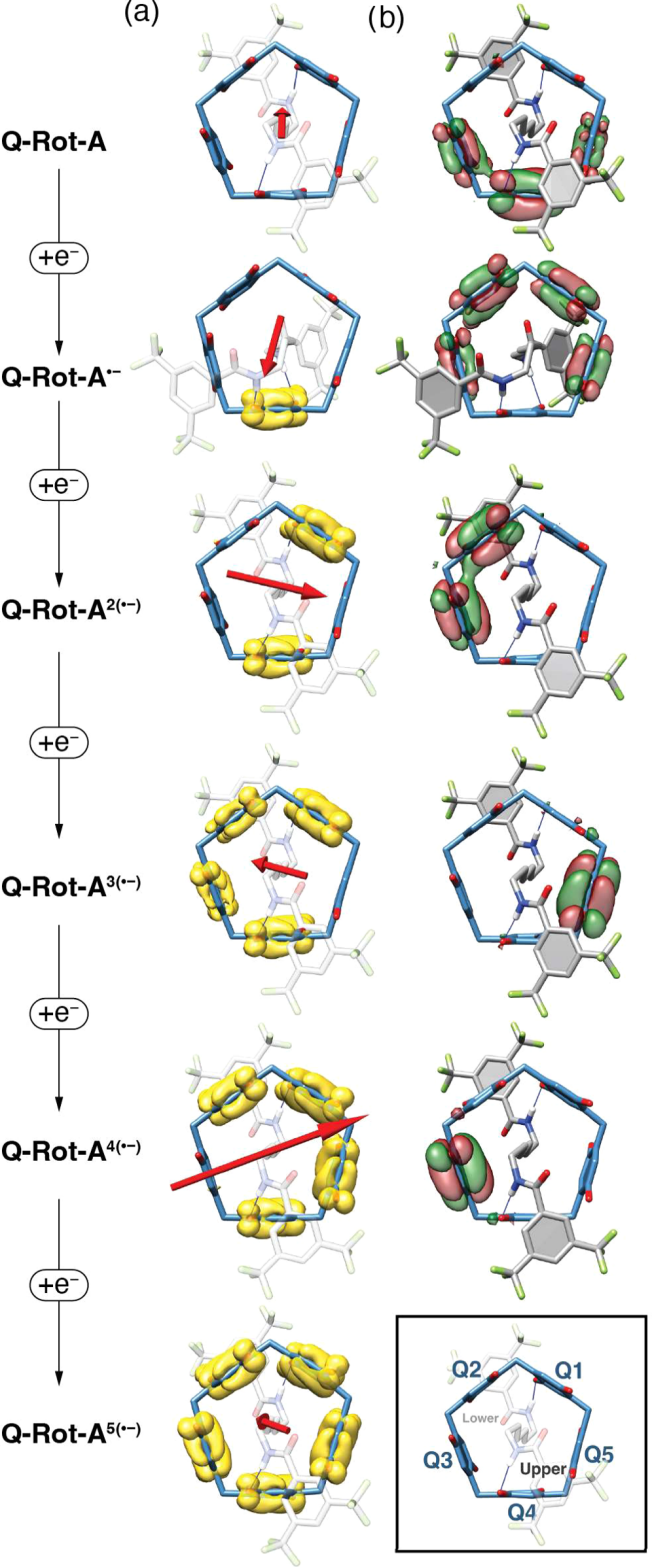
Spin density and LUMO plots for Q-Rot-A and its reduced species. (a) Spin density distributions for each species are shown in yellow with an isovalue of 0.0024. Red arrows represent the molecular dipole moments. (b) The lowest unoccupied molecular orbitals (LUMOs) for each species are depicted with isovalues of ±0.024. Inset: notation of the quinoid units and orientations (upper and lower) of the amide groups, consistent with structures depicted in Figure 8.

In order to gain deeper insight into the compass operation of **Q-Rot-A** at the molecular level, quantum chemical density functional theory (DFT) calculations were performed^24^ at the UM062*X*/6–31+G(d,p) level. Our initial focus was to determine the reduction sequence of quinoid units for **Q-Rot-A**, which corresponds to its experimentally observed 1–1–1–1–1 electron-uptake pattern (Figure 6a). For this purpose, we computed electron spin density distribution (yellow orbitals in Figure 7a) across the five quinoid units, labeled Q1–Q5 (the inset of Figure 7). The results revealed a stepwise reduction sequence: Q4 → Q1 → Q2/Q3 → Q5 → Q3. These findings support the arguments of Kaifer et al.^4^ and our own observations, indicating that quinoid units experiencing weaker electrostatic repulsions upon reduction are more prone to reduction.

The reduction sequence of Q4 → Q1 → Q2/Q3 → Q5 → Q3 can be further validated by determining the direction of the positive electrostatic potential. This direction means the pathway along which electrons are preferentially attracted, rendering the quinoid unit positioned in that orientation the most susceptible to reduction. In this context, the dipole moment serves as a reliable indicator of the electric field’s direction, with the arrow tail representing the positive charge and the arrowhead denoting the negative charge. In Figure 7a, red arrows illustrate the dipole moments for each reduced species of **Q-Rot-A**. These arrows consistently show that the positive end (arrow tail) points to the most reducible quinoid unit at each step of the reduction sequence: Q4, Q1, Q2/Q3, Q5, and Q3 for **Q-Rot-A**, **Q-Rot-A**^•**–**^, **Q-Rot-A**^**2(**•**−)**^, **Q-Rot-A**^**3(**•**−)**^, and **Q-Rot-A**^**4(**•**−)**^, respectively.

It is intriguing that spin delocalization occurs across Q2^*n*(•−)^ and Q3^*m*(•−)^ (*n* + *m* = 1, 0 < *n*, *m* < 1) for **Q-Rot-A**^**3(**•**−)**^ (Figure 7a). This phenomenon can, however, be understood by considering that electrons are delocalized through space between quinoid units, as confirmed by the anisotropy of the induced current density (AICD) calculations of free pillar[5]quinone^7b^ and all reduced species (Figure S15) of **Q-Rot-A**. Therefore, this observation raises an even more noteworthy question: why are radical electrons not delocalized across multiple quinoid units in other reduced species, such as **Q-Rot-A**^•**–**^, **Q-Rot-A**^**2(**•**−)**^, and **Q-Rot-A**^**4(**•**−)**^?

In order to shed light on the electronic states of these reduced species in relation to delocalization, the lowest unoccupied molecular orbitals (LUMOs) were scrutinized, as they are the most energetically favorable orbitals for reduction. Figure 7b depicts the LUMOs of each reduced species of **Q-Rot-A**. Notably, **Q-Rot-A**, and **Q-Rot-A**^•**–**^, and **Q-Rot-A**^**2(**•**−)**^ display LUMOs extended across multiple quinoid units, suggesting that an electron introduced via reduction would be delocalized throughout these quinoid units. Contrary to this exception, however, an added electron is not delocalized in the LUMO for **Q-Rot-A** and **Q-Rot-A**^•**–**^. Instead, the electron is localized to a single quinoid unit, specifically Q4^**•–**^ and Q1^**•–**^ (Figure 7a) for **Q-Rot-A**^•**–**^ and **Q-Rot-A**^**2(**•**−)**^, respectively. We attribute the disparity between the LUMOs and the spin density distribution to the spatial rearrangement of the dumbbell unit. When an electron is added to the LUMO of **Q-Rot-A** during its reduction, one side of the dumbbell component undergoes rotation (see the structural change between **Q-Rot-A** and **Q-Rot-A**^•**–**^ in Figure 7) to form two hydrogen bonds with Q4^•–^, stabilizing the localized spin electron at Q4^•–^. Likewise, in **Q-Rot-A**^•**–**^, the electron addition generates a negative charge on Q1 and prompts the lower part of the dumbbell component to rotate back toward Q1^•–^, forming a hydrogen bond that stabilizes and facilitates the localization of the spin electron at Q1^•–^. In contrast to these two cases, when **Q-Rot-A**^**2(**•**−)**^ is reduced, the dumbbell component maintains its original hydrogen bonds with Q1^•–^ and Q4^•–^, preserving its LUMO energy level. This subtle structural change allows the spin electron to reside in the unchanged LUMO, becoming delocalized across Q2^*n*(•−)^ and Q3^*m*(•−)^ in **Q-Rot-A**^**3(**•**−)**^. A distinct scenario emerges during the reduction of **Q-Rot-A**^**3 (**•**−)**^ to **Q-Rot-A**^**4(**•**−)**^, where the spin electron, initially delocalized over Q2^*n*(•−)^ and Q3^*m*(•−)^ in **Q-Rot-A**^**3(**•**−)**^, shifts to localize at Q2^•–^ in **Q-Rot-A**^**4(**•**−)**^. This electronic change is also driven by the rearrangement of the dumbbell unit. The emergence of a negative charge on Q5 in **Q-Rot-A**^**4(**•**−)**^ repels the C=O bond of the upper amide group away from Q2, consequently stabilizing the electron at Q2 more effectively than at Q3. The dynamic of the dipole moment pointers (amide groups) is explored in greater detail in the discussion below.

The molecule **Q-Rot-A** features two amide groups, each characterized by a strong dipole moment. These amide groups function like the pointer of a molecular compass, dynamically reorienting in response to changes in the reduction state of the quinoid units. Thus, the **Q-Rot-A** molecular system can be described as possessing two dipole moment pointers, which electrostatically tune the LUMO energies of the molecules. This modulation governs key electronic properties, including the sequence of reduction events and the extent of electron delocalization in the reduced species. A thorough understanding of the interplay between these dipole moment pointers and specific quinoid units is therefore vital for unraveling the reduction behavior of this molecular compass system.

**Figure 8:**
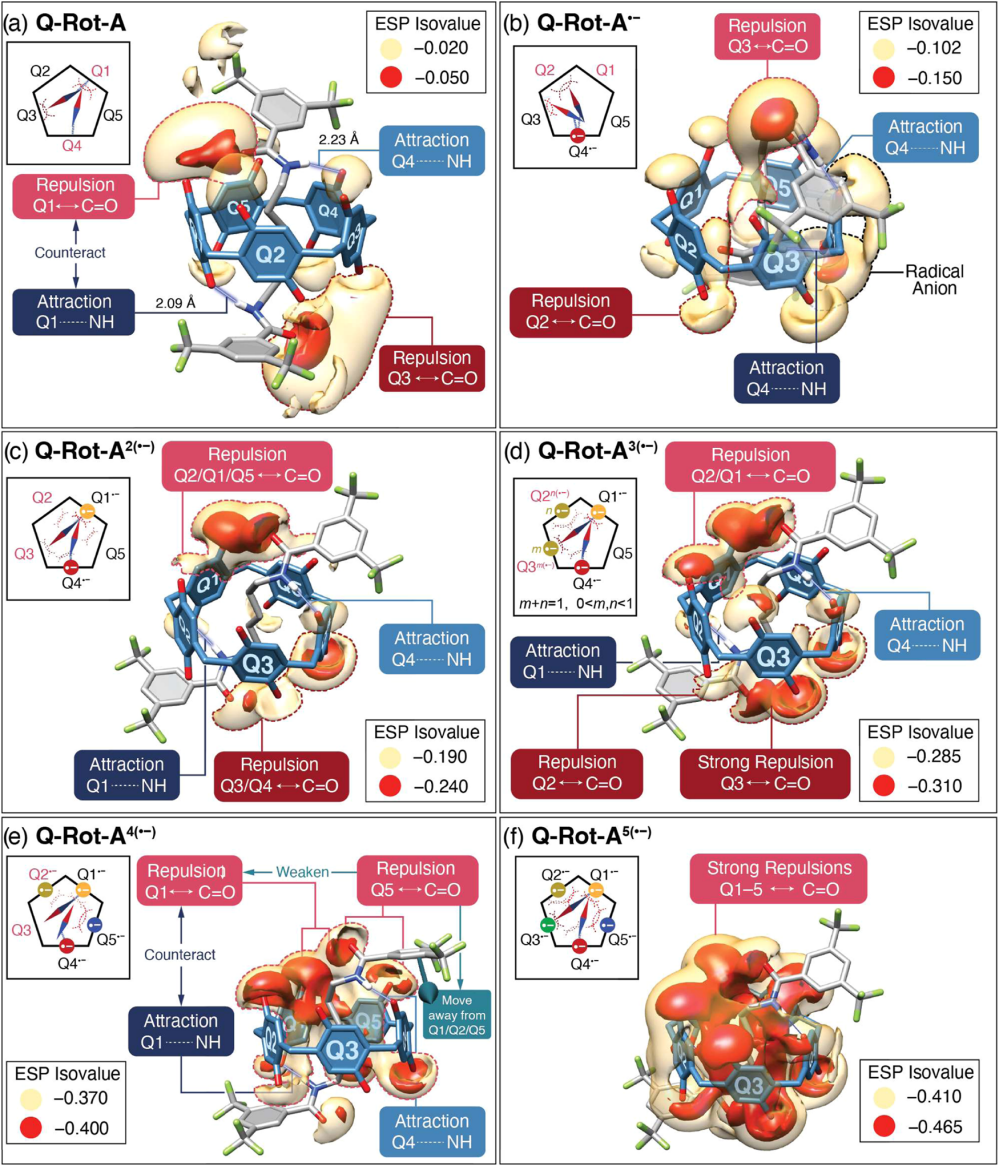
Electrostatic potential (ESP) isosurfaces and compass diagrams for (a) Q-Rot-A, (b) Q-Rot-A^•–^, (c) Q-Rot-A^2(•−)^, (d) Q-Rot-A^3(•−)^, (e) Q-Rot-A^4(•−)^, and (f) Q-Rot-A^5(•−)^. The red ESP isosurfaces represent regions of lower electrostatic potential compared to the yellow ESP isosurfaces, while areas beyond yellow isosurfaces indicate higher electrostatic potentials that exceed the isovalues of the yellow isosurfaces. In the compass diagrams, the pointer with brighter red and blue represents the upper amide group, whereas the one with darker red and blue represents the lower amide group in the molecular structure. The blue end (NH bond) carries a positive partial charge, and the red end (C=O bond) carries a negative partial charge. Blue and red dotted lines denote hydrogen bonds and repulsive electrostatic interactions, respectively.

In the case of **Q-Rot-A**, the upper and the lower dipole moment pointers have hydrogen bonds with Q1 and Q4, respectively, as illustrated in Figure 8a. It is evident that the favorable reduction (Figure 7a) of Q4 is associated with the electron-withdrawing effect of the hydrogen bonding, which is corroborated by experimental results for **Q-Rot-A** and **Q-Rot- E** in Figure 6a. It is noteworthy, however, that Q1 has a stronger hydrogen bond (*d*_H···O_: 2.087 Å) than Q4 (*d*_H···O_: 2.232 Å), yet exhibits a lower electrostatic potential (Figure S16) than Q4—in other words, Q1 is less reductive than Q4 despite its stronger hydrogen bonding. This discrepancy suggests the presence of repulsive interactions with Q1, which counteract the increase in electrostatic potential that would otherwise be enhanced by hydrogen bonding. In order to discern these repulsive interactions, the electrostatic potential (ESP) isosurfaces were depicted (Figure 8a) at isovalues of −0.020 (yellow) and −0.050 (red), overlaid on the molecular structure of **Q-Rot-A**. These ESP isosurfaces are predominantly situated around electronegative atoms, such as oxygen and fluorine. Notably, the ESP isosurfaces near the C=O bonds in dipole moment pointers extend to the region of the quinoid units, providing information about which quinoid unit is affected by these C=O bonds. For example, the red ESP isosurface (a brighter red dotted line in Figure 8a) between Q1 and the upper C=O bond suggests repulsive electrostatic interactions between the upper dipole moment pointer and Q1. These repulsive interactions play a critical role in decreasing the electrostatic potential of Q1 and compromising the enhancement induced by hydrogen bonding with the lower dipole moment pointer. On the other hand, the yellow ESP isosurface (a darker red dotted line in Figure 8a) between Q3 and the lower C=O bond indicates that the repulsive interactions with the lower dipole moment pointer decreases the electrostatic potential (Figure S16) of Q3 to some degree. All of these interactions of the dipole moment pointers are illustrated in its compass diagram (the inset of Figure 8a).

In the study of **Q-Rot-A**^•**–**^, we concentrated on identifying interactions involving the dipole moment pointers with Q1 and Q2 to decipher the reasons behind the higher reduction potential (Figure 6a) of Q1 relative to Q2. We directed our attention to Q1 and Q2 over Q3 and Q5, as the electrostatic potentials of Q3 and Q5 are decreased mainly by electrostatic repulsions from the negative charge of Q4^•–^, rather than by interactions with the dipole moment pointers. A notable structural change during the reduction of **Q-Rot-A** to **Q-Rot-A**^•**–**^ is the reorientation of the dipole moment pointers, as illustrated in its compass diagram (the inset of Figure 8b). The negative charge on Q4^•–^ induces strong attractions toward the NH bonds in the dipole moment pointers, driving the two C=O bonds to shift away from Q4^•–^. In order to gain in-depth understanding of the electrostatic landscape surrounding these C=O bonds, the ESP isosurfaces are visualized at isovalues of −0.102 (yellow) and −0.150 (red) in Figure 8b. Three overlapping isosurfaces are discerned in the lower region of Q2, the upper region of Q3, and vicinity of Q4^•–^. First, the yellow and red ESP isosurfaces (a black dotted line in Figure 8b) surrounding Q4^•–^ delineate the negative electrostatic potentials of the spin electron localized at Q4^•–^. Second, the yellow ESP isosurface (a brighter red dotted line in Figure 8b), positioned between Q3 and the upper C=O bond, indicates the presence of repulsive interactions between Q3 and the upper dipole moment pointer. These interactions contribute to the diminished electrostatic potential (Figure S16) of Q3 compared to that of Q5. Lastly, the most significant feature is the yellow ESP isosurface (a darker red dotted line in Figure 8b) between Q2 and the lower C=O bond, which indicates that the lower dipole moment pointer has a repulsive effect on Q2. These interactions account for the reduced electrostatic potential (Figure S16) of Q2 compared to that of Q1 in **Q-Rot-A**^•**–**^, making Q1 more reductive.

To understand the compass operation of **Q-Rot-A**^**2(**•**−)**^, we centered our analysis on the interactions between the dipole moment pointers and Q2/Q3. This focus stems from the fact that the reduction of Q5 is primarily hindered by electrostatic repulsions from radical anions on Q1^•–^ and Q4^•–^, rather than interactions with the dipole moment pointers. During the reduction of **Q-Rot-A**^•**–**^ to **Q-Rot-A**^**2(**•**−)**^, the most significant structural change involves the rotation of the C=O bond in the lower amide group, which reorients from Q4^•–^ to Q1^•–^ and forms a hydrogen bond with Q1^•–^ (Figure 8c). Concurrently, the upper amide group sustains its original hydrogen bonding with Q4^•–^. These alignments of the dipole moments are further reinforced by multiple noncovalent interactions involving the stoppers, including lone pair–π interactions, hydrogen-bonding, π–π stacking, and multipolar interactions, as demonstrated by the independent gradient model (IGM) analysis (Figure S17). Consequently, the C=O bond in the upper dipole moment pointer experiences repulsive interactions with Q1^•–^, Q2, and Q5, as shown by the yellow ESP isosurface (a brighter red dotted line in Figure 8c). In contrast, the C=O bond in the lower dipole moment pointer reduces the electrostatic potentials (a darker red dotted line in Figure 8c) of Q3 and Q4^•–^. Coincidentally, the upper and lower dipole moment pointers have similar impacts on Q2 and Q3, respectively, leading to a balanced distribution of the LUMO across Q2 and Q3 in **Q-Rot-A**^**2(**•**−)**^ (Figure 7b).

In the reduction of **Q-Rot-A**^**2(**•**−)**^ to **Q-Rot-A**^**3(**•**−)**^, the added electron in **Q-Rot-A**^**3(**•**−)**^ is delocalized across Q2^*n*(•−)^ and Q3^*m*(•−)^, as evidenced by their comparable spin densities (Figure 7a). This delocalization indicates that the balanced influence of the dipole moment pointers on Q2 and Q3 in **Q-Rot-A**^**2(**•**−)**^ persists even in **Q-Rot-A**^**3(**•**−)**^. This balance is interesting because the emergence of negative charges on Q2^*n*(•−)^ and Q3^*m*(•−)^ in **Q-Rot-A**^**3(**•**−)**^ intensifies electrostatic repulsions, potentially altering the interplay between the quinoid units and the dipole moment pointers. To explore these interactions in greater detail, the ESP isosurfaces for **Q-Rot-A**^**3(**•**−)**^ are present in Figure 8d. Q2^*n*(•−)^ is influenced by the upper dipole moment pointer, as indicated by the yellow ESP isosurface (a brighter red dotted line in Figure 8d) between them. In comparison, the overlapping red ESP isosurface between Q3^*m*(•−)^ and the lower dipole moment pointer signifies significantly stronger repulsions between them. Nevertheless, the lower dipole moment pointer also reduces the electrostatic potential of Q2^*n*(•−)^, as shown by the yellow ESP isosurface (a darker red dotted line in Figure 8d) between them, thereby contributing to a balanced electronic level between Q2^*n*(•−)^ and Q3^*m*(•−)^.

When analyzing **Q-Rot-A**^**4(**•**−)**^, a significant observation is the localization of the unpaired electron at Q2^•–^ (Figure 7a), which was previously delocalized across Q2^*n*(•−)^ and Q3^*m*(•−)^ in **Q-Rot-A**^**3(**•**−)**^. This localization correlates with the displacement of the upper dipole moment pointer away from Q2^*n*(•−)^, as depicted in Figures 8e and S18. The resulting increase in the electrostatic potential of Q2^*n*(•−)^, coupled with the decrease in the electrostatic potential of Q3^*m*(•−)^, strengthens its attraction to the electron, resulting in a higher electron probability at Q2^*n*(•−)^. To investigate the cause of this displacement, we analyzed the interactions of the dipole moment pointers using ESP isosurfaces (Figure 8e). The yellow ESP isosurface (a brighter red dotted line in Figure 8e) between Q5^•–^ and the upper C=O bond reveals repulsive interactions that displace (Figure S18) the upper dipole moment pointer away from Q1^•–^, Q2^•–^, and Q5^•–^ toward the Q3. Consequently, the observation of a discontinuous red ESP isosurface between Q1^•–^ and the upper C=O bond (Figure 8e), in contrast to its connected state in **Q-Rot-A**^**3(**•**−)**^, indicates weakened repulsive interactions in this region. These reduced repulsions alter electronic levels, causing the electron to become localized at Q2^•–^.

In **Q-Rot-A**^**5(**•**−)**^, the C=O bonds in the dipole moment pointers exhibit strong electrostatic repulsions with the radical anions at all quinoid units (Figure 8f), as evidenced by the red ESP isosurface (isovalue: −0.465) spanning over the quinoid units. Importantly, the reduction of Q3 to Q3^•–^ in **Q-Rot-A**^**5(**•**−)**^ induces repulsion of the upper dipole moment pointer back toward Q5^•–^, leading to a structural alignment (Figure S18) similar to that of **Q-Rot-A**^**3(**•**−)**^. Furthermore, the yellow ESP isosurfaces (isovalue: −0.410) enveloping all quinoid units highlight the low negative electrostatic potential characteristic of the pentaradical pentaanion.

## Conclusions

We have designed [2]rotaxane-based molecular compasses, wherein the dipole moments of amide and ester groups within the dumbbell component serve as pointers to respond to electric field changes during the reduction of the pillar[5]quinone ring component. Our design leverages mechanical bonds to enable these dipole moment pointers to rotate at the center of the pillar[5]quinone’s cavity while maintaining their fixed positions within the cavity. However, synthesizing pillar[5]quinone-based [2]rotaxanes presents significant challenges due to the quinone moiety’s reactivity as a Michael acceptor, which precludes the direct use of pillar[5]quinone as a host in the threading-followed-by-stoppering method. Additionally, attempts to oxidize the 1,4-dimethoxybenzene units of permethylated pillar[5]arene-based [2]rotaxanes into *p*-benzoquinone units result in the degradation of the rotaxane structure. To overcome these obstacles, we developed an alternative strategy: we first synthesized permethylated pillar[5]arene-based [2]rotaxane, followed by the demethylation to yield a perhydroxylated pillar[5]arene-based [2]rotaxane. Subsequent oxidation of this perhydroxylated pillar[5]arene-based [2]rotaxane produced the desired pillar[5]quinone-based [2]rotaxane. This synthetic approach underscores the novelty of our efforts in overcoming the inherent reactivity challenges of pillar[5]quinone, ultimately enabling the successful construction of these mechanically interlocked molecular systems.

The reductive behavior of pillar[5]quinone-based [2]rotaxanes, which function as molecular compasses, differs from that of free pillar[5]quinone, despite both incorporating the pillar[5]quinone structure. This difference arises primarily from the dipole moment pointers, which enable electronic communication between remote quinoid units by conveying information about their reduction states. To elucidate the role and interactions of these dipole pointers, we employed a suite of density functional theory (DFT)-based computational methods, including geometry optimization, spin density distribution analysis, dipole moment evaluation, LUMO energy calculations, independent gradient model (IGM) analysis, and electrostatic potential (ESP) mapping with isosurface visualization. These investigations reveal that hydrogen bonding via NH groups and repulsive electrostatic interactions from C=O bonds critically govern the reduction characteristics—namely, the reduction sequence, electron delocalization, and electronic communication—by modulating the electrostatic potentials of individual quinoid units. These findings illuminate the intricate structural-electronic dynamics underpinning the functionality of these mechanically interlocked molecular systems. Our prototypical investigation on using the directionality of a molecular compass to modulate the electronic interplay between redox sites could offer valuable insights into new molecular device designs, electric field and polarity probing of various materials, electrostatic interactions in biomolecules, and innovative synthetic methods such as selective functionalization.

## Supporting Information Available

Experimental details (chemical information, synthetic protocols, experimental conditions, and parameters) and characterization (NMR spectroscopy, CD spectroscopy, and electrochemical measurements) (PDF)
