# Supplementary material for: Molecular Compasses for Modulating Electronic Communication in Pillar[5]quinone
Source: J Am Chem Soc. 2025 Apr 5;147(15):12425–37. doi: 10.1021/jacs.4c12280 (PMC12006990; doi:10.1021/jacs.4c12280)
Supplement: Supplementary file 1 — ja4c12280_si_001.pdf [file ja4c12280_si_001.pdf]

Molecular Compasses for Modulating Electronic Communication in Pillar[5]quinone

Tae-woo Kwon,[1,*] Guangcheng Wu,[1] Sheng-Nan Lei[1] and J. Fraser Stoddart[1,2,3,4,5,6,▿]

[1] *Department of Chemistry, The University of Hong Kong, Hong Kong SAR 999077, China*
[2] *Department of Chemistry, Northwestern University, 2145 Sheridan Road, Evanston, Illinois 60208, United States*
[3] *Center for Regenerative Nanomedicine, Northwestern University, Chicago, 60611, United States*
[4] *Stoddart Institute of Molecular Science, Department of Chemistry, Zhejiang University, Hangzhou 310027, China*
[5] *ZJU-Hangzhou Global Scientific and Technological Innovation Center, Hangzhou 311215, China*
[6] *School of Chemistry, University of New South Wales, Sydney, NSW 2052, Australia*
[▿] *Deceased on December 30, 2024.*

* E-mail: taewoo@hku.hk

Supporting Information

Table of Contents

Section A. General Information **S2**

Section B. Synthetic Protocols **S3**

Section C. NMR Spectroscopy **S8**

Section D. ¹H DOSY NMR Spectroscopy **S18**

Section E. Circular Dichroism Spectroscopy **S20**

Section F. Electrochemical Measurements **S21**

Section G. Computational Studies **S22**

Section H. References **S26**

Section A. General Information

All commercially available reagents and solvents were purchased from Sigma Aldrich and they were used as received without further purification. Permethylated pillar[5]arene (**DMP5A**) was synthesized by following previously reported procedures.[1, 2] Normal-phase liquid chromatography was performed on a Combiflash Rf 200 purification system, using FlashPure EcoFlex Silica (Büchi). Nuclear magnetic resonance (NMR) spectra were recorded at 298 K on a Bruker Avance III 500 MHz spectrometer, with a working frequency of 500 MHz for ^{1}H NMR spectra as well as 125 MHz for ^{13}C NMR spectra. The 2D ^{1}H-^{1}H NOESY spectra were recorded on a Bruker Avance III 500 MHz spectrometer with a mixing time of 0.3 seconds. Diffusion ordered spectroscopy (DOSY) experiments were performed at 298 K in CD_2Cl_2 at a typical concentration of 1 mg mL^{-1} on a Bruker Avance III 600 MHz spectrometer at a frequency of 600 MHz. The variable temperature NMR spectroscopy was performed on a Bruker Avance III 600 MHz spectrometer. The gradient strength was incremented in 16 steps from 2 to 95% of the maximum gradient strength. Chemical shifts are reported in ppm relative to the signals corresponding to the residual non-deuterated solvents ($CDCl_3$: δ 7.26 ppm for ^{1}H and 77.16 ppm for ^{13}C). High-resolution mass spectra (HRMS) were measured on an Agilent 6545 Quadrupole Time-of-Flight LC-MS, using an ESI source. X-Ray crystallographic data were obtained on a XtaLAB Synergy diffractometer equipped with a micro-focus sealed X-ray tube PhotonJet (Cu) X-ray. All electrochemical experiments were performed at room temperature using a glassy carbon working electrode (0.071 cm^2) in N_2-purged solutions of dry $CHCl_3$ with a Gamry Multipurpose instrument (Reference 600). The electrode surface was polished routinely with 0.05 μm alumina-water slurry on a felt surface immediately before use. We used platinum wire (Pt) as the counter electrode and Ag/AgCl as the reference electrode. The sample and supporting electrolyte ($TBAPF_6$) in $CHCl_3$ had concentrations of 1.0 mM and 0.1 M, respectively. Normal pulse voltammetry (NPV) experiments were performed with a step size of 5 mV, a sample period of 0.1 ms, and a pulse time of 0.05 s. Square wave voltammetry (SWV) was also performed from 0.15 to −2 V with a pulse size of 20 mV and a frequency of 15Hz.

Section B. Synthetic Protocols

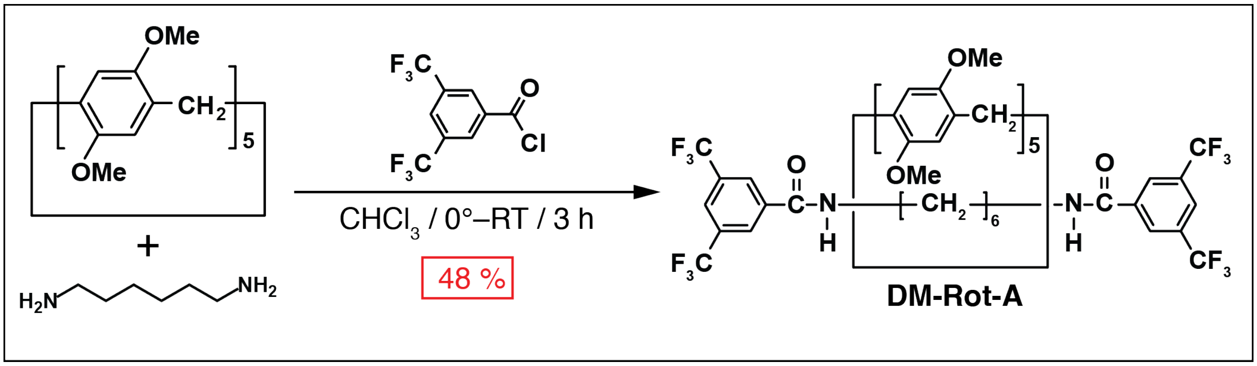

Scheme S1. Synthesis of **DM-Rot-A**

DM-Rot-A (Scheme S1): DMP5A (1.0 g, 0.186 mmol) and 1,6-diaminohexane (186 mg, 1.598 mmol) were dissolved in anhydrous $CHCl_3$ (27 mL). The solution was cooled down to 0 °C in an ice bath. A solution of 3,5-bis(trifluoromethyl)benzoyl chloride (724 μL) in anhydrous $CHCl_3$ (7 mL) was added slowly to the first solution. The reaction mixture was stirred at room temperature for 3 h, during which time the solution temperature naturally increased to room temperature. After MeOH (80 mL) was added, a formed precipitate, which was confirmed to be free **DMP5A**, was filtered off and washed with additional MeOH (10 mL). The filtrate was evaporated, and the solids were washed with MeOH, filtered, and dried under reduced pressure to afford **DM-Rot-A** as a white solid (958 mg, 48 %). The ^{1}H NMR spectrum was consistent with that for **DM-Rot-A** reported in the previous literature.[2] ^{1}H NMR ($CDCl_3$, 500 MHz, 298 K): δ 8.40 (s, 4H), 8.09 (s, 2H), 6.92 (s, 10H), 6.52 (s, 2H), 3.76 (s, 10H), 3.70 (s, 30H), 2.44 (m, 2H), 2.22 (m, 2H), –0.61 (m, 4H), –1.62 (m, 4H).

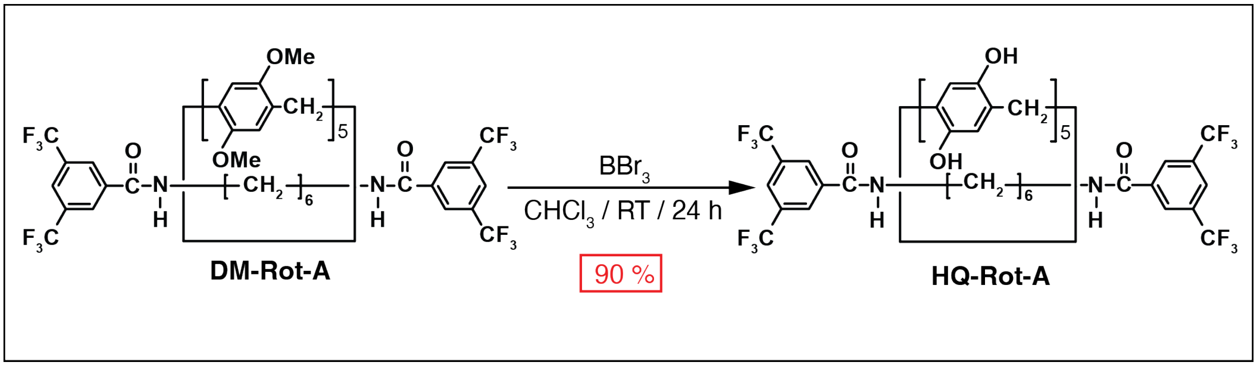

Scheme S2. Synthesis of **HQ-Rot-A**

HQ-Rot-A (Scheme S2): DM-Rot-A (849 mg, 0.630 mmol) was stirred in anhydrous $CHCl_3$ (18 mL). Boron tribromide (1.215 mL, 12.607 mmol) was added to the solution. The reaction vessel was sealed with a glass stopper. After the reaction mixture was stirred at 25°C for 24 h, MeOH was slowly added into the mixture to quench the reaction until it stopped fuming. After the solvent was evaporated, MeOH (18 mL) was added. 0.5 M aqueous HCl (36 mL) was added to precipitate the product. The solution was stirred overnight. The precipitate was filtered, washed with H_2O and $CHCl_3$, and dried under reduced pressure to afford **HQ-Rot-A** as a white solid (682 mg, 90%). ^{1}H NMR (CD_3COCD_3, 500 MHz, 298 K): δ 8.66 (s, 4H), 8.31 (s, 2H), 7.00 (s, 2H), 6.69 (s, 2H), 6.64 (s, 2H), 6.61 (s, 4H), 3.97 (d, J = 13.6 Hz, 2H), 3.95 (d, J = 13.6 Hz, 2H), 3.65 (s, 2H), 3.20 (d, J = 13.6 Hz, 2H), 3.14 (d, J = 13.6 Hz, 2H), 2.66–2.57 (m, 2H), 2.56–2.48 (m, 2H), –0.04 (s, 4H), –1.26 (s, 4H). ^{13}C NMR (CD_3COCD_3, 125 MHz, 298 K): δ 165.5, 149.2, 147.9, 147.6, 147.4, 146.3, 137.9, 132.8, 132.5, 132.24, 132.0, 129.1, 129.1, 128.7, 128.1, 128.0, 127.9, 127.5, 126.5, 125.9, 125.4, 123.2, 121.0, 119.2, 118.4, 118.4, 117.5, 41.6, 30.9, 30.8, 29.1, 25.1. HRMS-ESI m/z 1207.3252 ($[M + H]^+$, $C_{59}H_{50}F_{12}N_2O_{12}$ calc. 1207.3245)

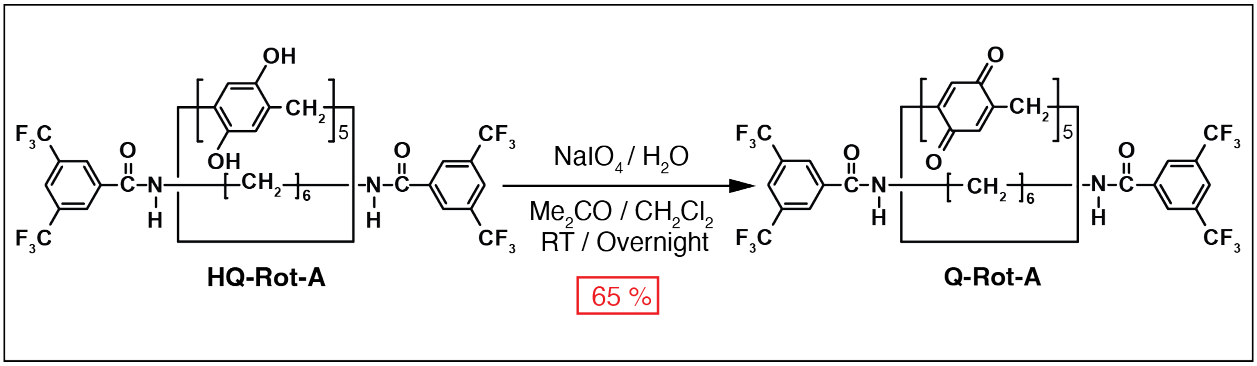

Scheme S3. Synthesis of **Q-Rot-A**

Q-Rot-A (Scheme S3): HQ-Rot-A (260 mg, 0.215 mmol) was stirred in Me_2CO (1 mL) and CH_2Cl_2 (1 mL). After aqueous solution (507 mg in 3.7 mL of H_2O) of $NaIO_4$ was added, the reaction mixture was stirred at room temperature overnight. The organic layer was separated, and the aqueous layer was washed with CH_2Cl_2. The collected organic phase was dried ($MgSO_4$) and filtered. The filtrate was evaporated, and the crude mixture was recrystallized from hexane (50 mL) and CH_2Cl_2 to afford **Q-Rot-A** as a yellow solid (169 mg, 65 %). ^{1}H NMR ($CDCl_3$, 500 MHz, 298 K) δ 8.45 (s, 4H), 8.06 (s, 2H), 6.77 (s, 10H), 6.19 (s, 2H), 3.49 (s, 10H), 2.78–2.67 (m, 2H), 2.62–2.51 (m, 2H), 0.52–0.37 (m, 4H), 0.20–−0.02 (m, 4H). ^{13}C NMR ($CDCl_3$, 125 MHz, 298 K) δ 186.6, 164.0, 143.6, 136.3, 135.9, 132.2, 132.0, 128.0, 126.5, 125.1, 124.4, 122.2, 120.0, 39.9, 29.6, 26.9, 26.7. HRMS-ESI *m/z* 1197.2490 ([M+H]$^+$, $C_{59}H_{40}F_{12}N_2O_{12}$ calc. 1197.2462)

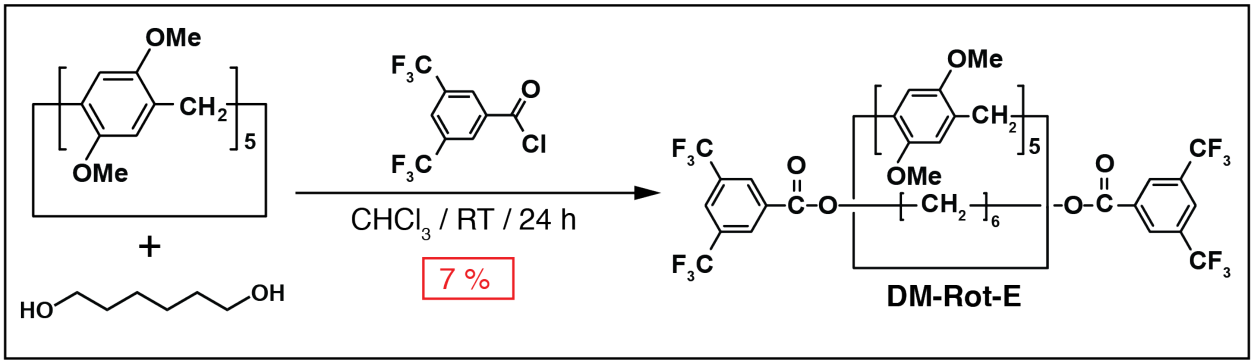

Scheme S4. Synthesis of **DM-Rot-E**

DM-Rot-E (Scheme S4): DMP5A (1.4 g, 1.86 mmol) and 1,6-hexanediol (264 mg, 2.24 mmol) were stirred in anhydrous $CHCl_3$ (28 mL) at room temperature. After confirming the materials were completely dissolved, $NaHCO_3$ (940 mg, 11.19 mmol) and 3,5-bis(trifluoromethyl)benzoyl chloride (1.014 mL, 5.59 mmol) were added. The reaction mixture was then stirred for 24 h at room temperature. Insoluble solids were removed by filtration, and the filtrate was extracted with brine. The collected organic phase was dried over anhydrous Na_2SO_4 and evaporated under reduced pressure. The resulting solids were dissolved in hexane. Insoluble solids, confirmed to be pure *per*-methylated pillar[5]arene, were filtered off, and the filtrate was dried under reduced pressure. The dried solids were stirred in MeOH (50 mL), collected by filtration, and purified by SiO_2 column chromatography (EtOAc/Hex = 0 to 5 %) to afford **DM-Rot-E** as a white powder (179 mg, yield: 7%). ^{1}H NMR ($CDCl_3$, 500 MHz, 298 K) δ 8.50 (s, 4H), 8.14 (s, 2H), 6.94 (s, 10H), 3.79 (s, 10H), 3.76 (s, 30H), 3.56–3.38 (m, 4H), 0.03–−0.11 (m, 4H), −2.20–−2.32 (m, 4H)

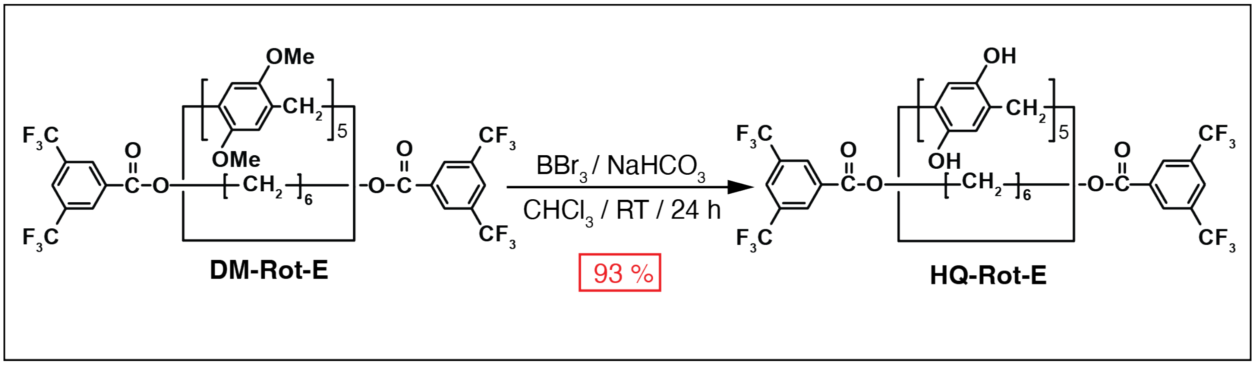

Scheme S5. Synthesis of **HQ-Rot-E**

HQ-Rot-E (Scheme S5): DM-Rot-A (68 mg, 0.050 mmol) and NaHCO3 (254 mg, 3.024 mmol) were stirred in BBr_3 solution (1.0 mL, 1 M in CH_2Cl_2). The reaction vessel was sealed with a glass stopper. After stirring the reaction mixture at 25 °C for 24 h, H_2O was slowly added to quench the reaction until fuming ceased. The organic phase was collected and dried under reduced pressure, yielding **HQ-Rot-E** as a white solid (93%). Without further characterization, **Q-Rot-E** was synthesized using **HQ-Rot-E**, as **HQ-Rot-E** is easily oxidized in air.

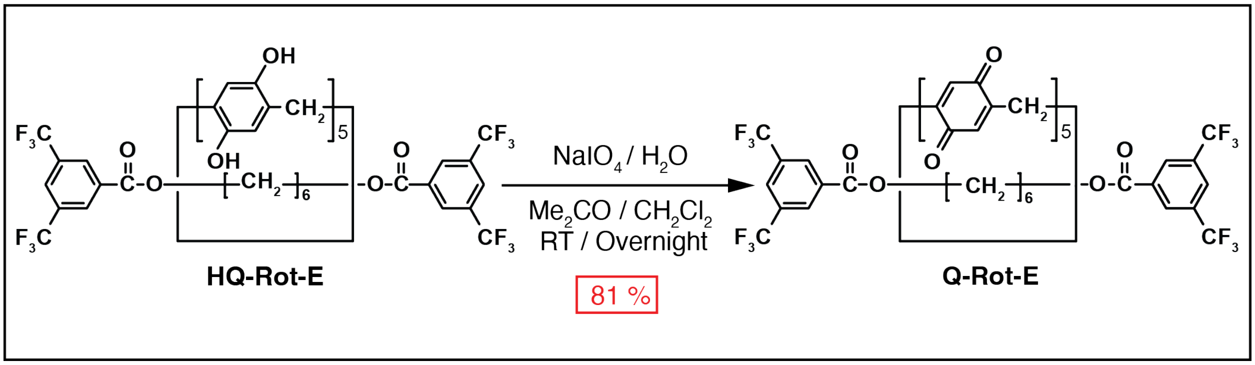

Scheme S6. Synthesis of **Q-Rot-E**

Q-Rot-E (Scheme S6): HQ-Rot-E (36 mg, 0.030 mmol) was stirred in Me_2CO (0.15 mL) and CH_2Cl_2 (0.3 mL). After $NaIO_4$ aqueous solution (70 mg in 0.51 mL of H_2O) was added, the reaction mixture was stirred at room temperature overnight. The organic phase was separated, and the aqueous phase was washed with CH_2Cl_2 three times. The collected organic phased was dried over $MgSO_4$, filtered, and dried under reduced pressure to afford **Q-Rot-E** as a yellow solid (29 mg, Yield: 81 %) ^1H NMR ($CDCl_3$, 500 MHz, 298 K) δ 8.44 (s, 4H), 8.13 (s, 2H), 6.74 (s, 10H), 3.67–3.53 (m, 4H), 3.47 (s, 10H), 0.93–0.78 (m, 4H), 0.48–0.35 (m, 4H). ^{13}C NMR ($CDCl_3$, 125 MHz, 298 K) δ 186.4, 164.0, 143.5, 135.8, 132.6, 132.3, 132.1, 132.0, 131.8, 130.3, 126.6, 126.5, 124.3, 122.1, 119.9, 65.4, 29.9, 28.6, 26.6, 25.2.

Section C. NMR Spectroscopy

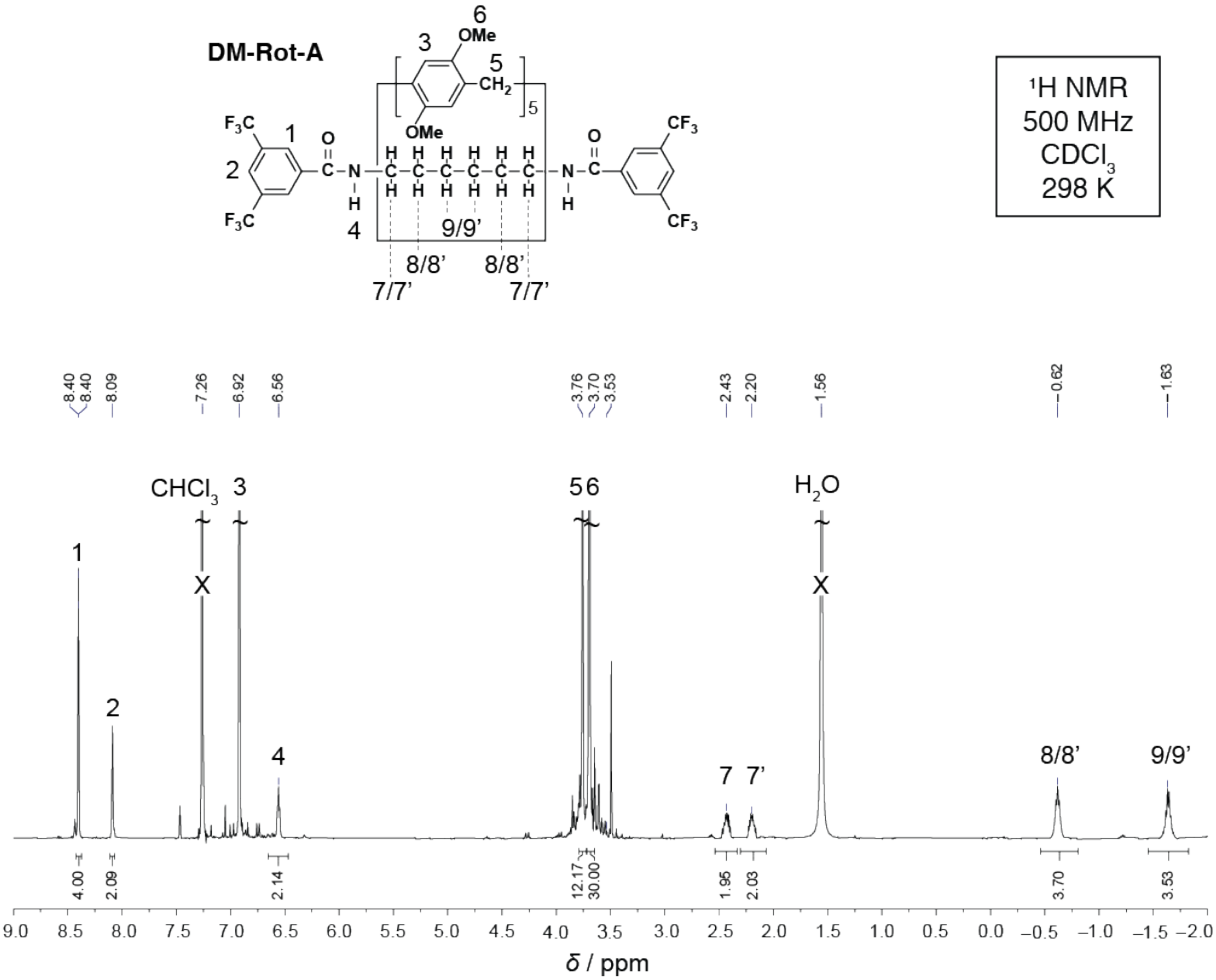

Figure S1. ¹H NMR Spectrum (500 MHz, $CDCl_3$, 298 K) of **DM-Rot-A**

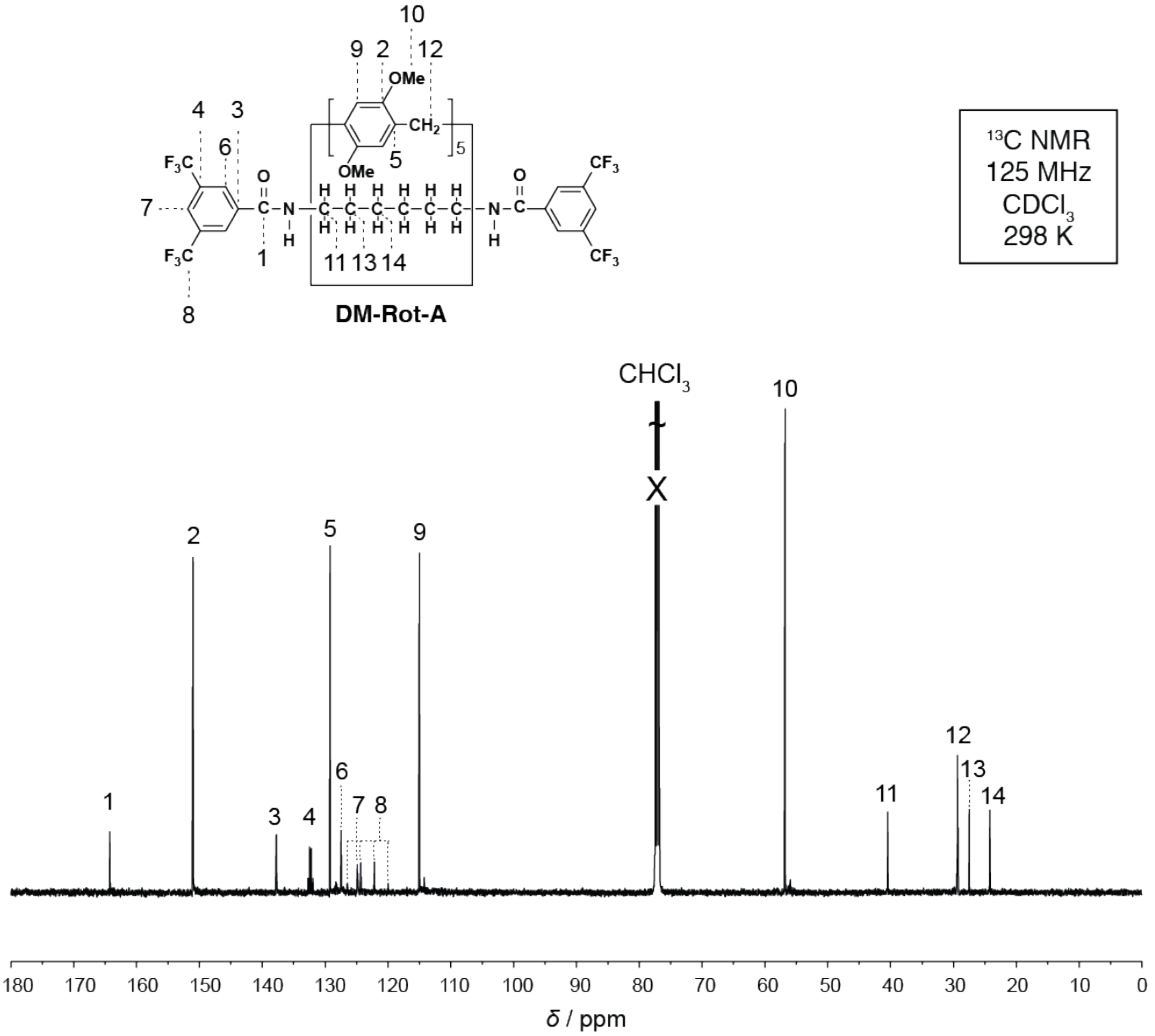

Figure S2. ^{13}C NMR Spectrum (125 MHz, $CDCl_3$, 298 K) of **DM-Rot-A**

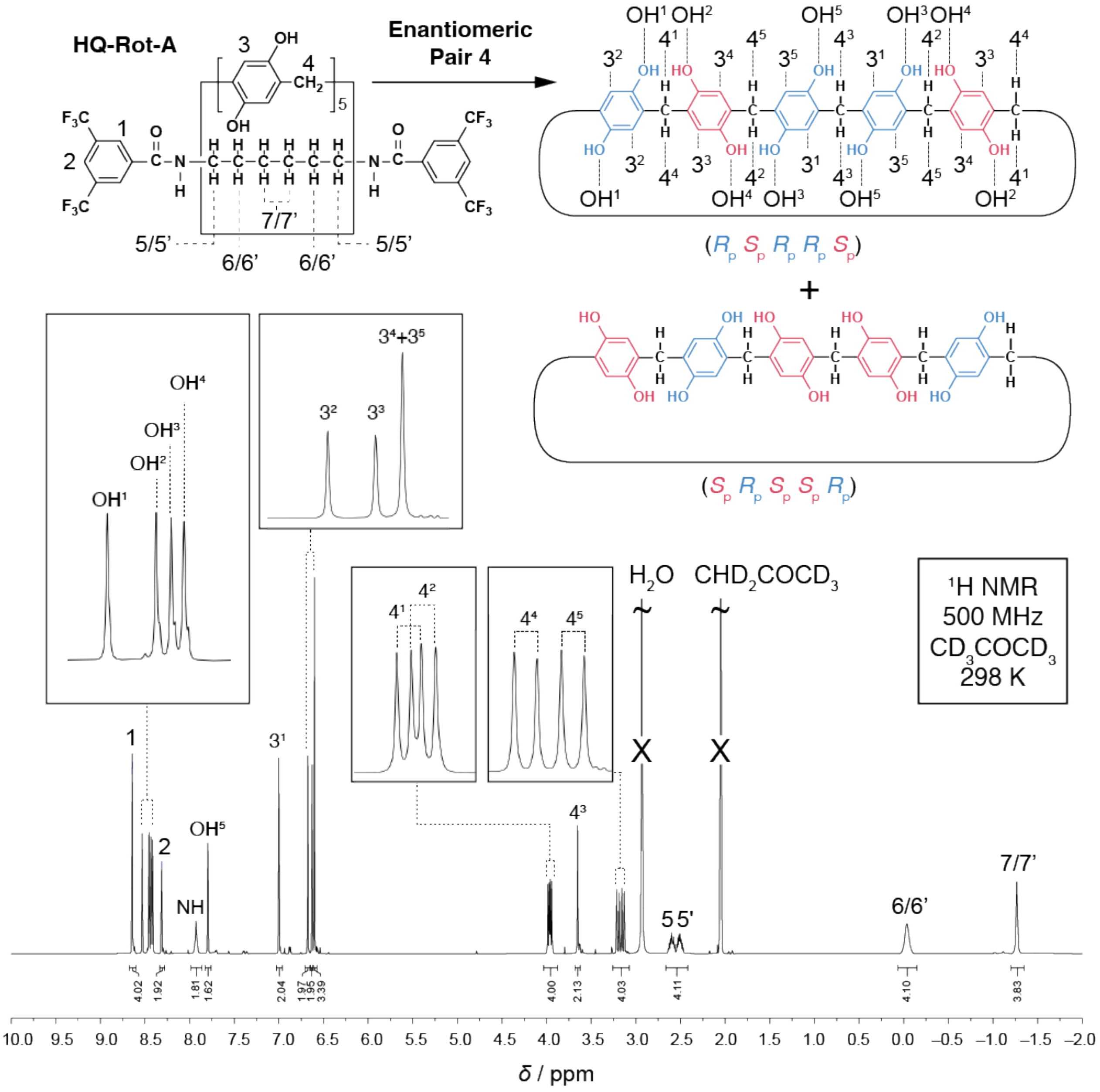

Figure S3. ^{1}H NMR Spectrum (500 MHz, CD_3COCD_3, 298 K) of **HQ-Rot-A**

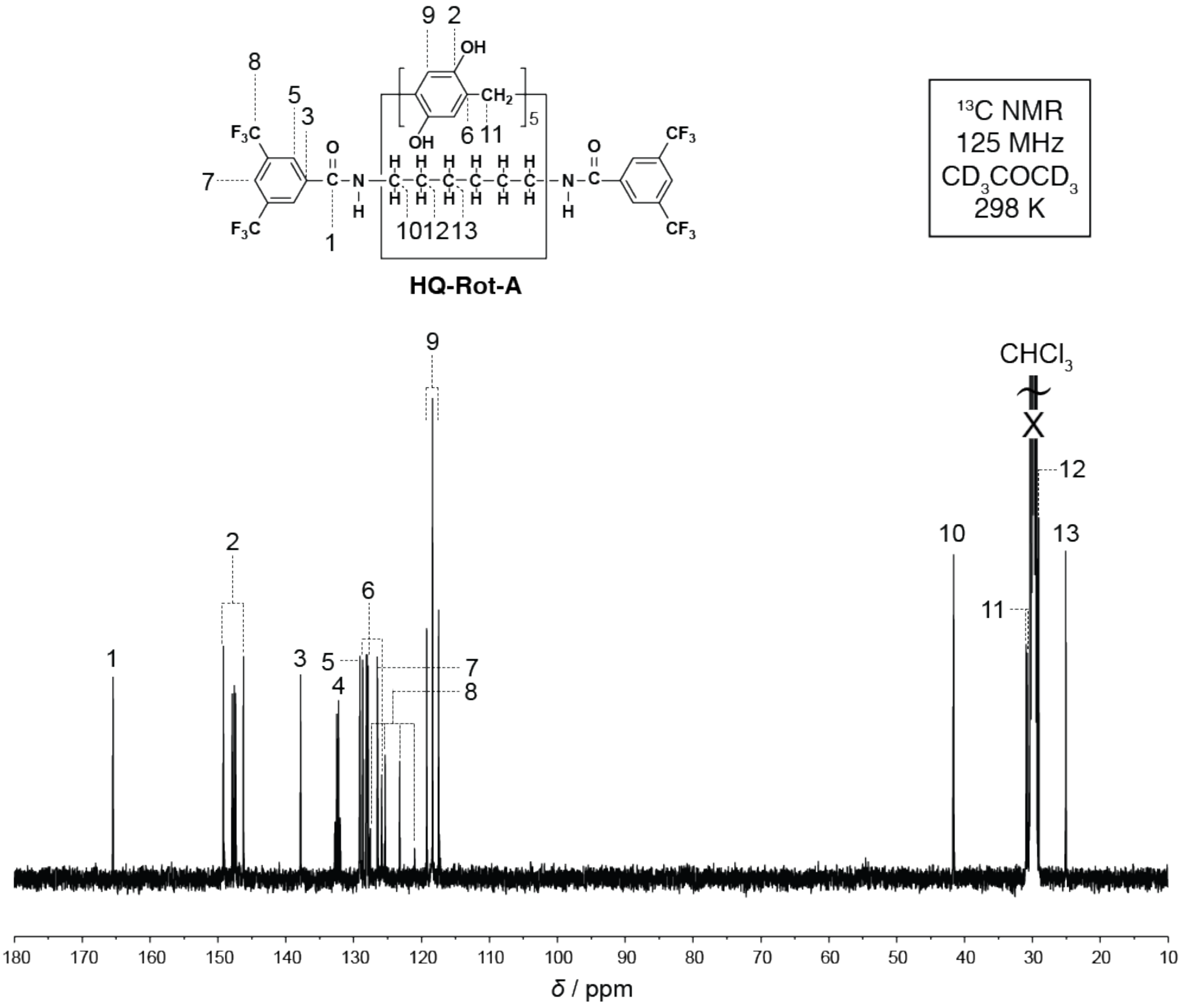

Figure S4. ^{13}C NMR Spectrum (125 MHz, CD_3COCD_3, 298 K) of **HQ-Rot-A**

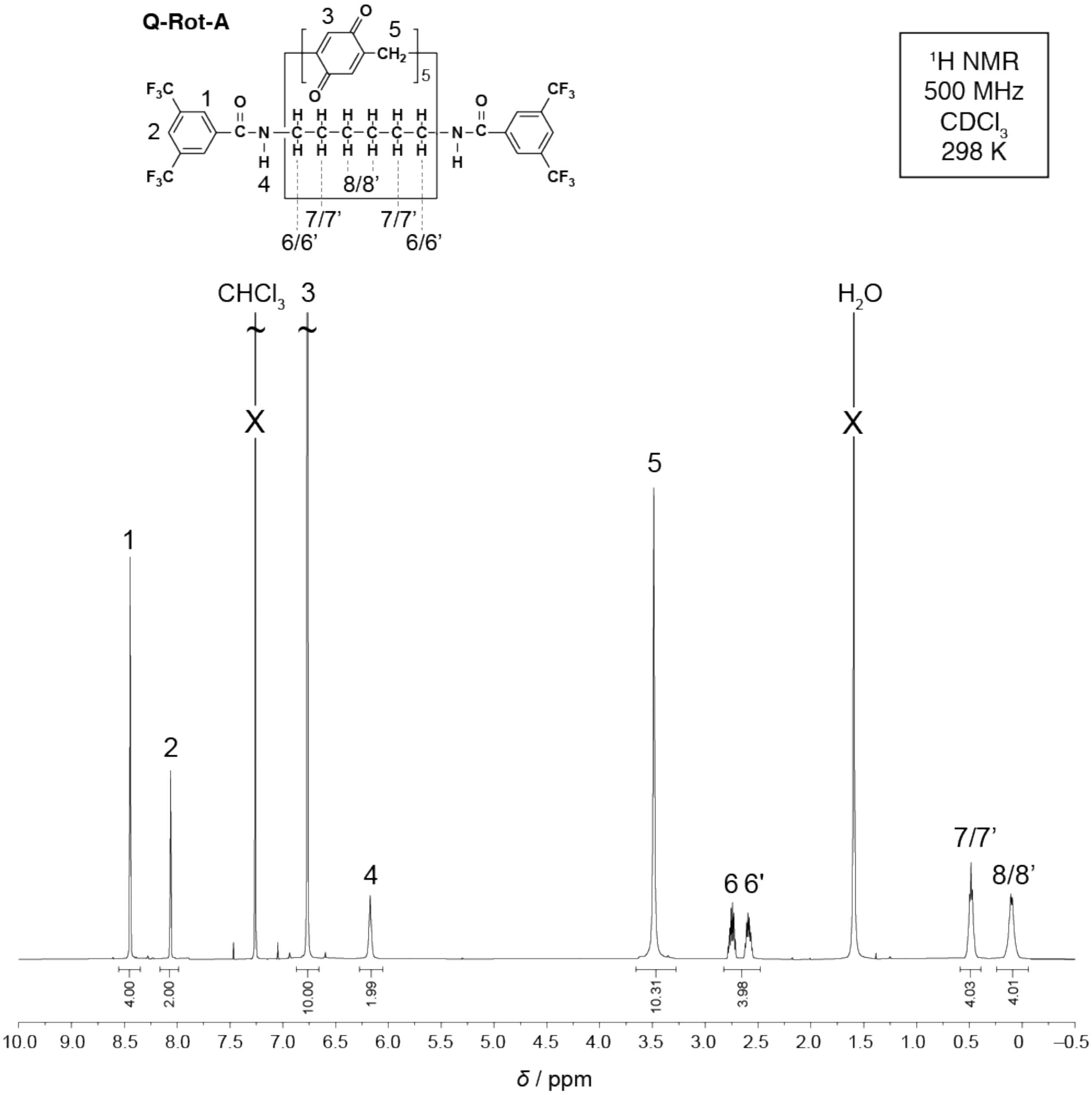

Figure S5. ^{1}H NMR Spectrum (500 MHz, $CDCl_3$, 298 K) of **Q-Rot-A**

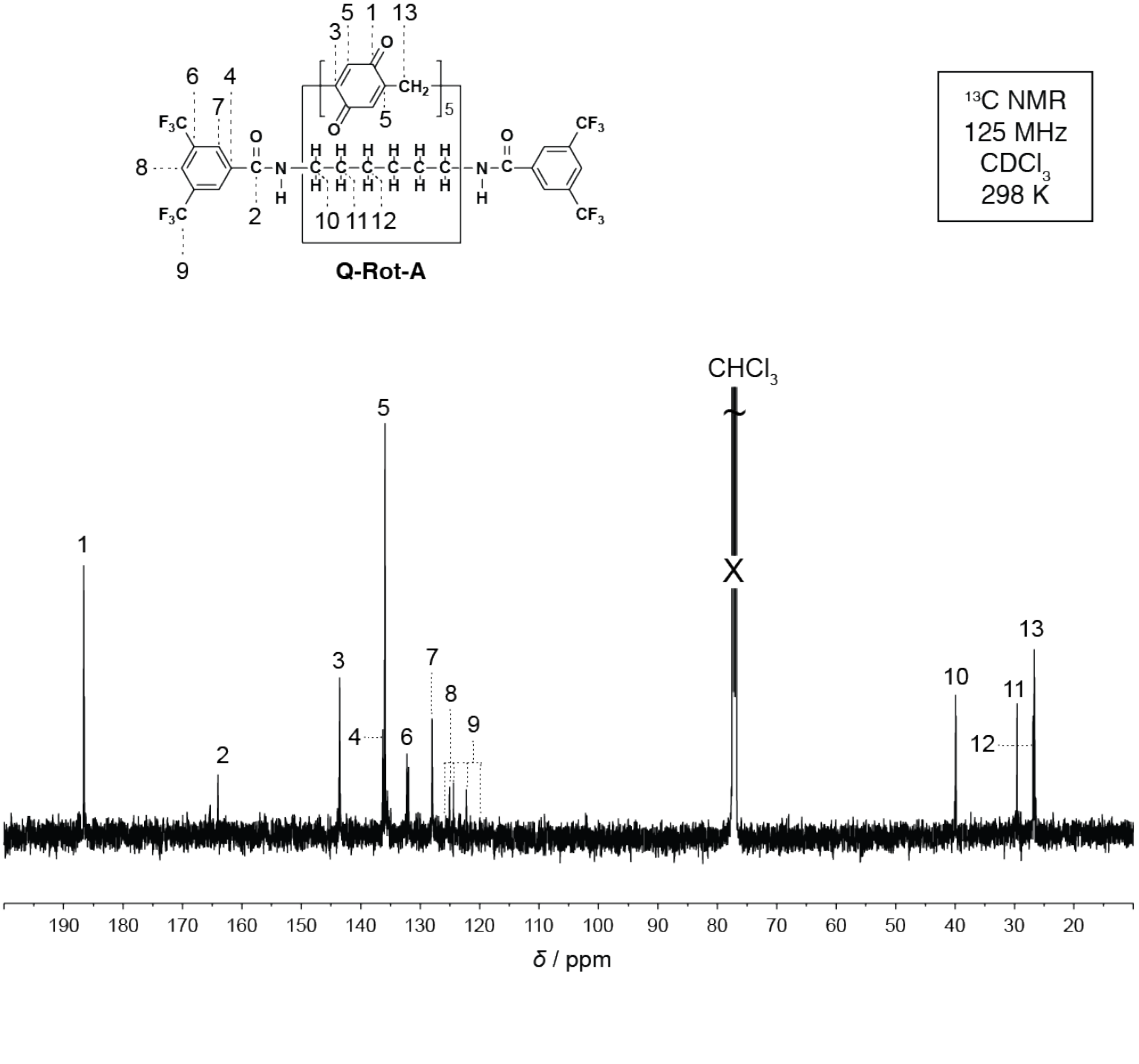

Figure S6. ^{13}C NMR Spectrum (125 MHz, $CDCl_3$, 298 K) of **Q-Rot-A**

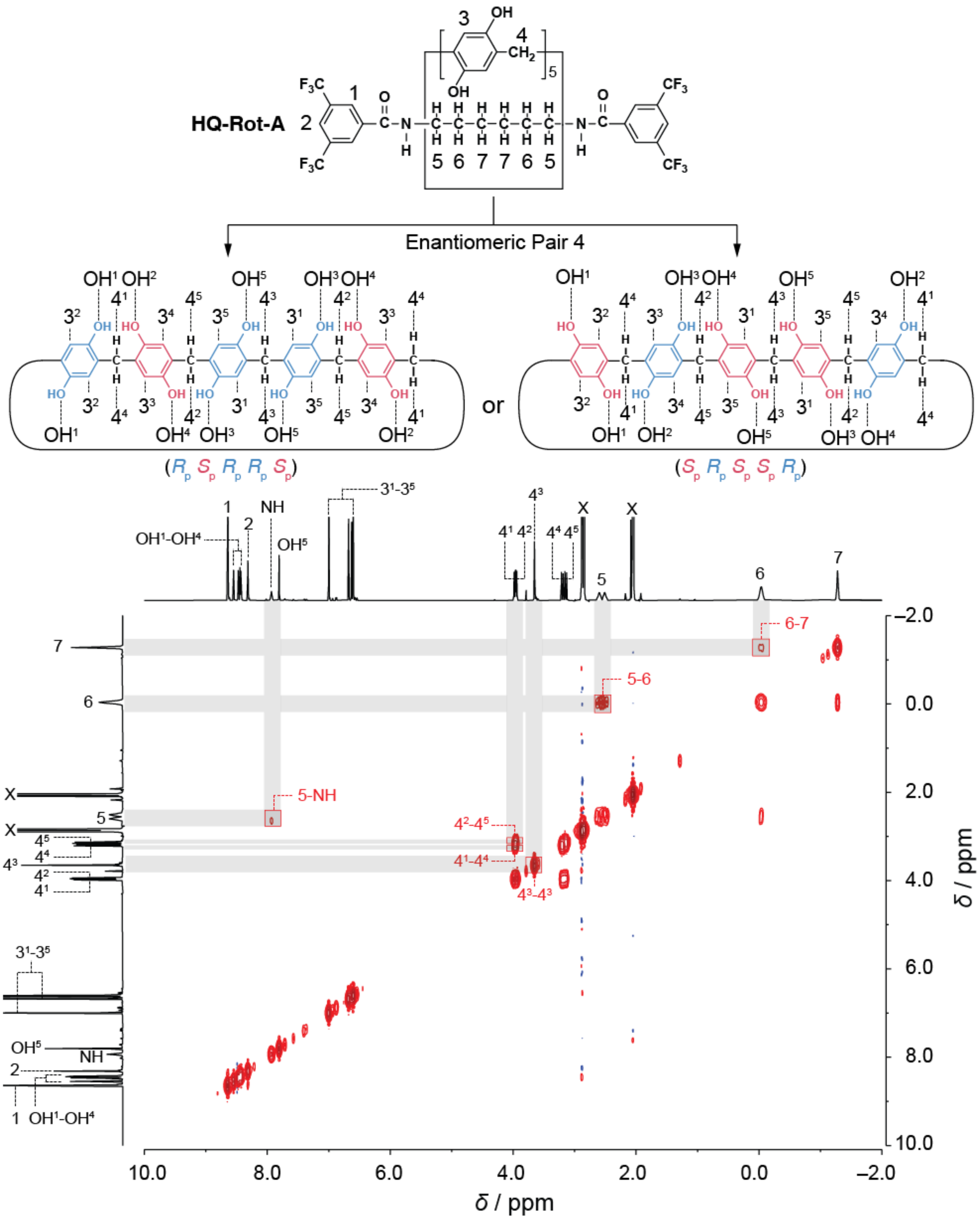

Figure S7. 2D ^{1}H-^{1}H COSY NMR Spectrum (500 MHz, $CDCl_3$, 298 K) of **HQ-Rot-A**. Cross peaks between protons 4^1 and 4^4, as well as between protons 4^2 and 4^5, indicate that these protons are geminal to each other. The fact that proton 4^3 has an only diagonal peak suggests that it has a homotopic relationship with another proton in the molecule.

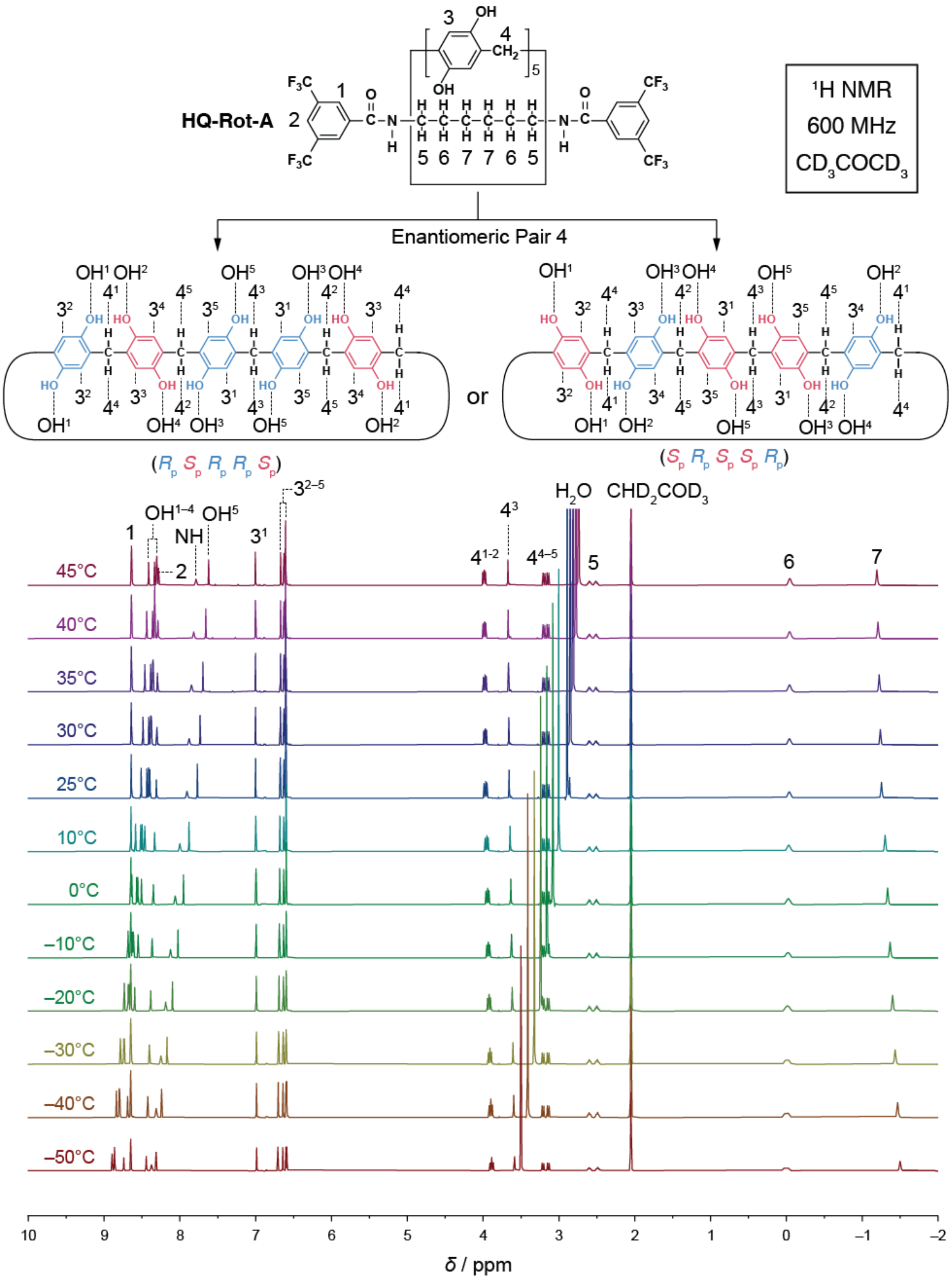

Figure S8. Variable temperature ^{1}H NMR spectra (600 MHz, CD_3COCD_3, 298K) of **HQ-Rot-A**

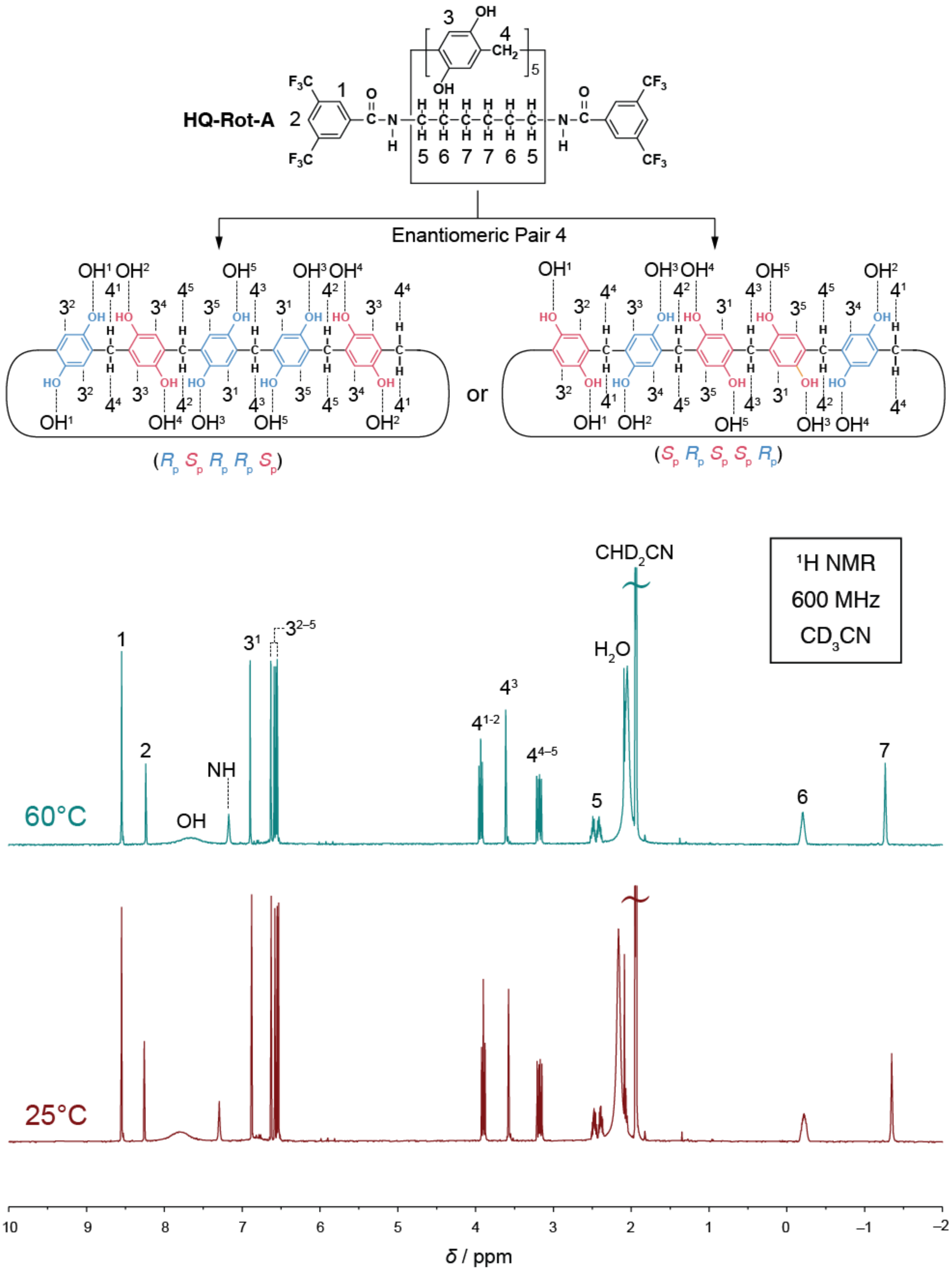

Figure S9. Variable temperature ^{1}H NMR spectra (600 MHz, CD_3CN) of **HQ-Rot-A**

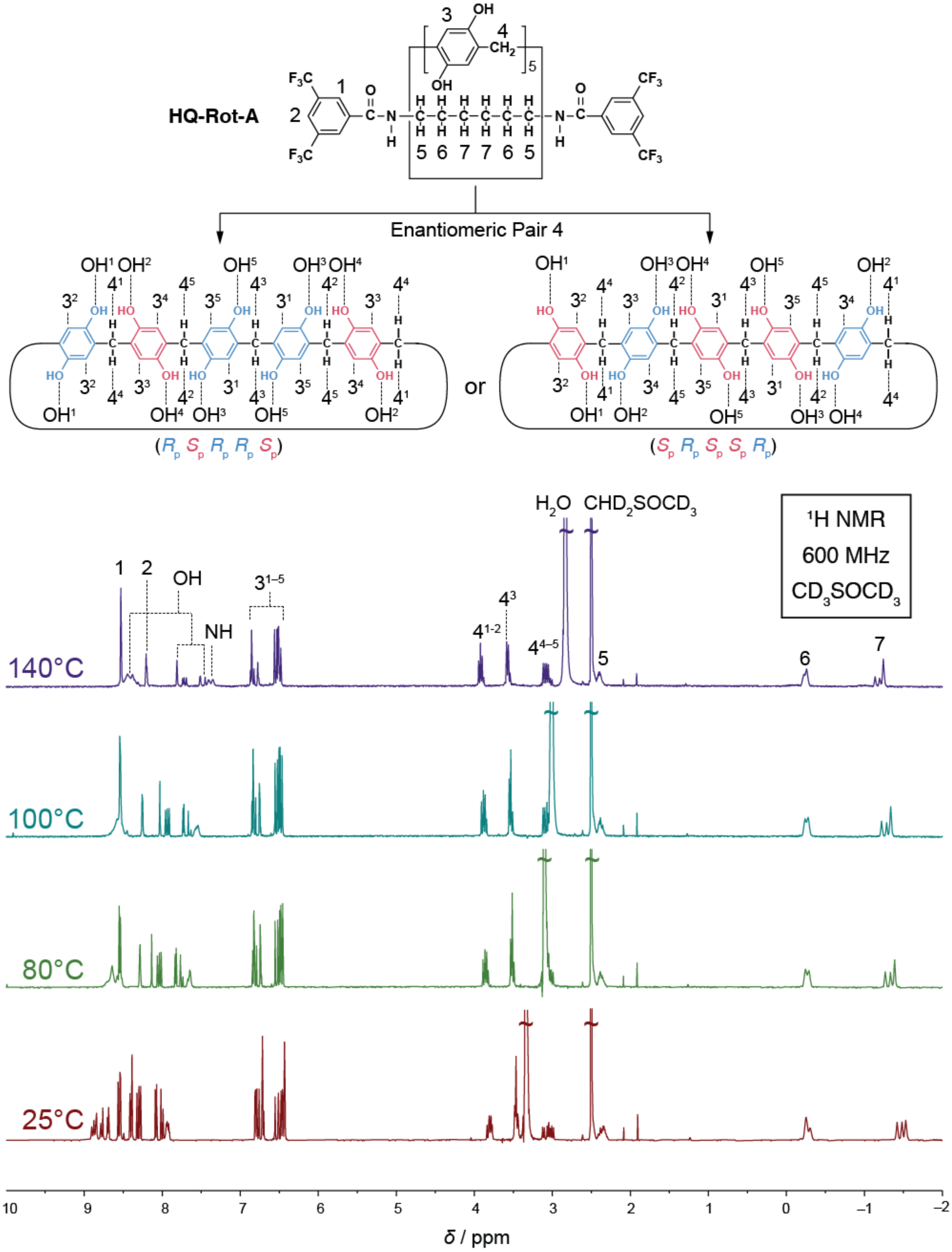

Figure S10. Variable temperature ^{1}H NMR spectra (600 MHz, CD_3SOCD_3) of **HQ-Rot-A**

Section D. ^{1}H DOSY NMR Spectra

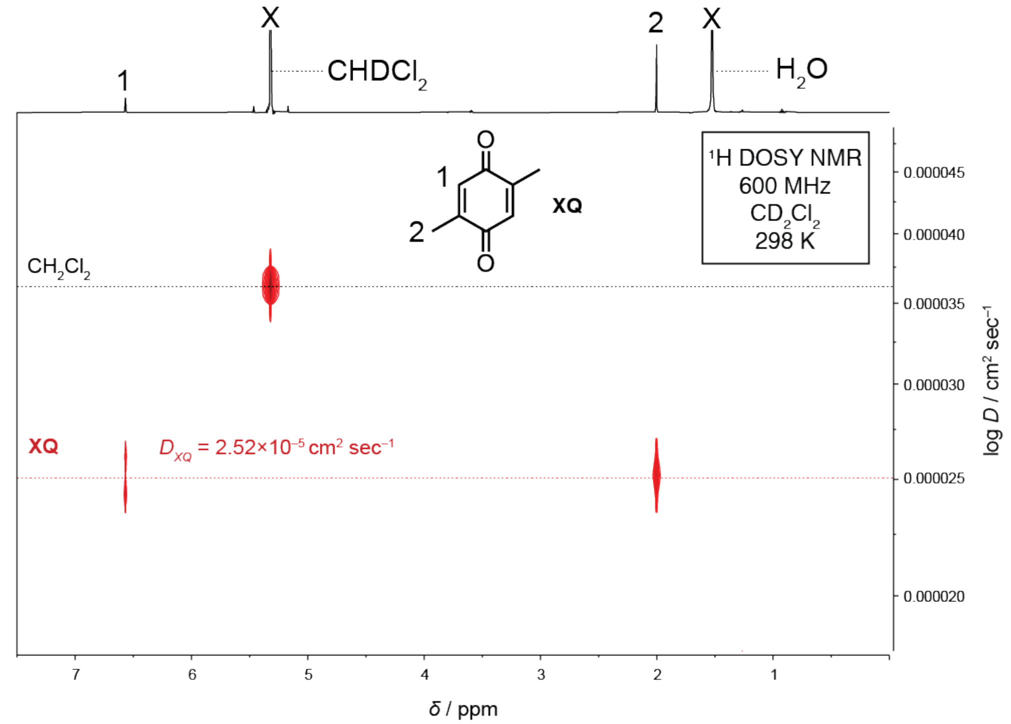

Figure S11. ^{1}H DOSY NMR Spectrum (600 MHz, CD_2Cl_2, 298K) of **XQ** (1 mM)

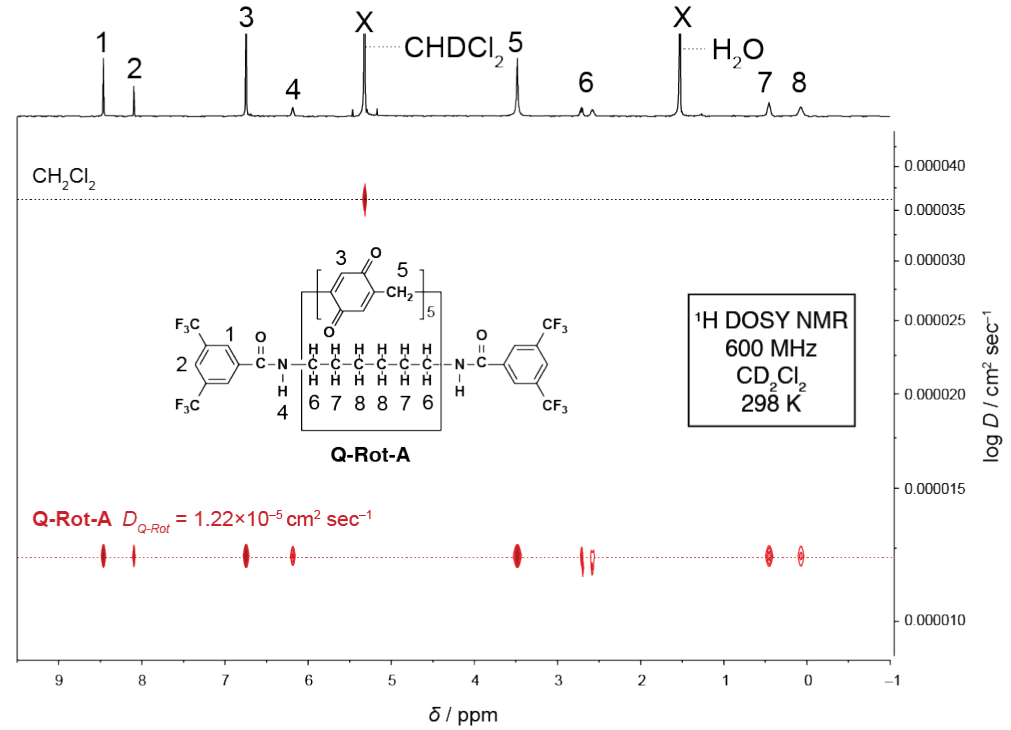

Figure S12. ^{1}H DOSY NMR Spectrum (600 MHz, CD_2Cl_2, 298K) of **Q-Rot-A** (1.0 mM)

We measured the diffusion coefficients (D) of **XQ** and **Q-Rot-A** (1.0 mM) in CD_2Cl_2 by ^{1}H DOSY NMR spectroscopy (Figure S11 and S12). The values were $D_{NMR,XQ} =$ 2.52×10^{-5} and $D_{NMR,Q-Rot} =$ 1.22×10^{-5} cm^2 s^{-1}, respectively. To calculate the diffusion coefficients in voltammetric cells containing **XQ** and **Q-Rot-A** (1.0 mM) in CD_2Cl_2 including 0.1 M $TBAPF_6$ as a supporting electrolyte, we used the Stokes-Einstein-Sutherland equation (equation S1) to adjust for the viscosity difference between the solutions.

$$r = \frac{kT}{6\pi\eta D} \qquad \text{Equation S1}$$

where r is the radius of the molecular sphere, k is the Boltzmann constant, T is the temperature in Kelvin, η is the viscosity of the medium, and D is the diffusion coefficient. If the same temperature (T) and the same molecule (r) are used for different two media, the Stokes-Einstein-Sutherland equation leads to equation S2.

$$D_{cell} = \frac{\eta_{NMR}}{\eta_{cell}} D_{NMR} \qquad \text{Equation S2}$$

We used an Ubbelohde viscometer to measure η_{NMR} (CD_2Cl_2) and η_{cell} (CH_2Cl_2 containing 0.1 M $TBAPF_6$). The viscosity (η) of the solution is calculated by the following equation:

$$\eta = B\rho t \qquad \text{Equation S3}$$

where B is a calibration constant that depends on the geometry of the viscometer, ρ is the density of the fluid, t is the measured flow time in the viscometer. We obtained the ratio of the viscosities of the NMR and cell solutions as $\frac{\eta_{NMR}}{\eta_{cell}} = 0.959$.

Based on the equation S2, we calculated the diffusion coefficients (D) of **XQ** and **Q-Rot-A** in the voltametric cells as $D_{cell,XQ} = 2.42 \times 10^{-5}$ and $D_{cell,Q-Rot-A} = 1.17 \times 10^{-5}\ cm^2\ S^{-1}$, respectively.

Section E. Circular Dichroism (CD) Spectroscopy

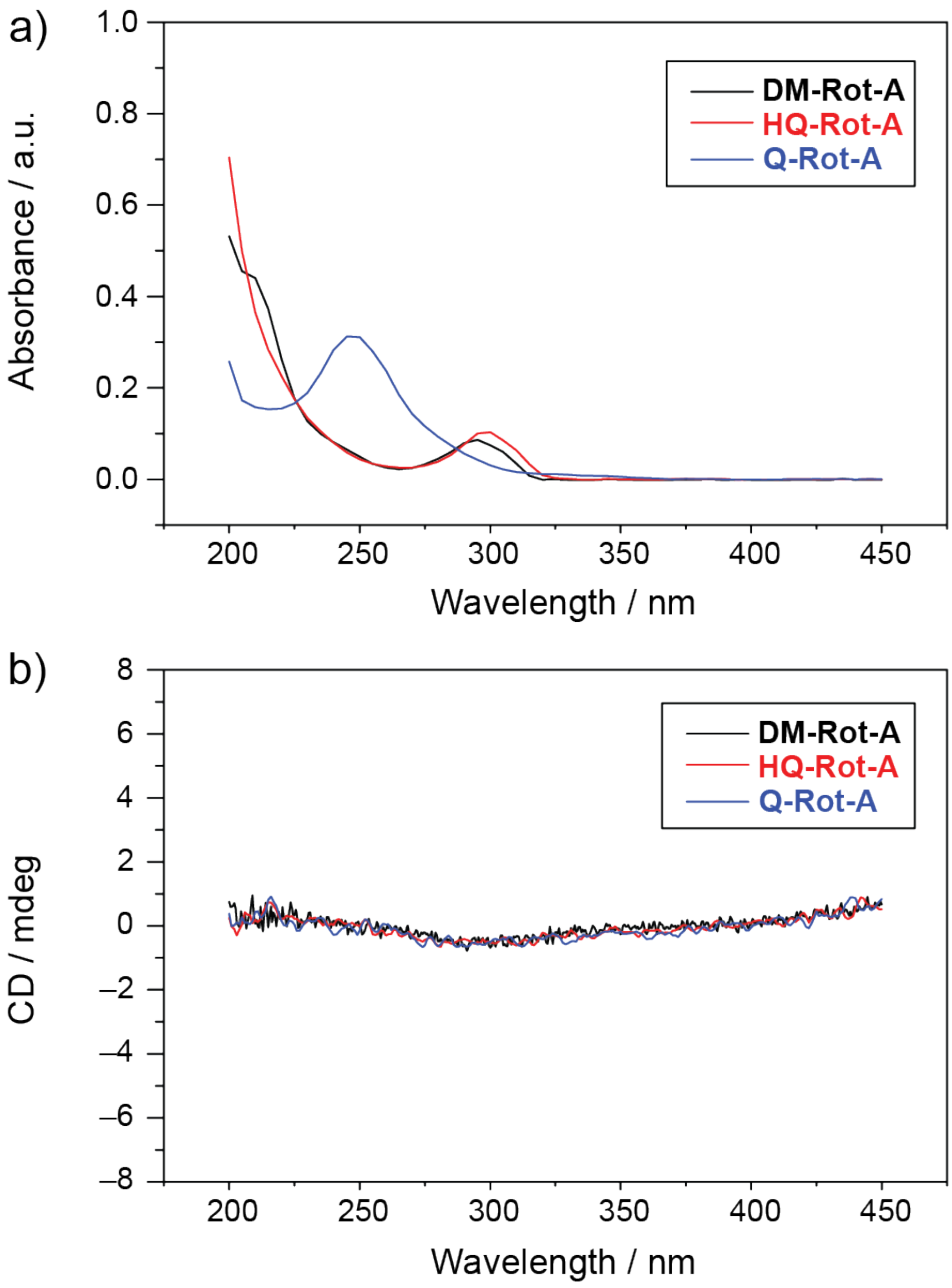

Figure S13. (a) Absorbance and (b) CD spectra of **DM-Rot-A**, **HQ-Rot-A**, and **Q-Rot-A**

Section F. Electrochemical Measurements

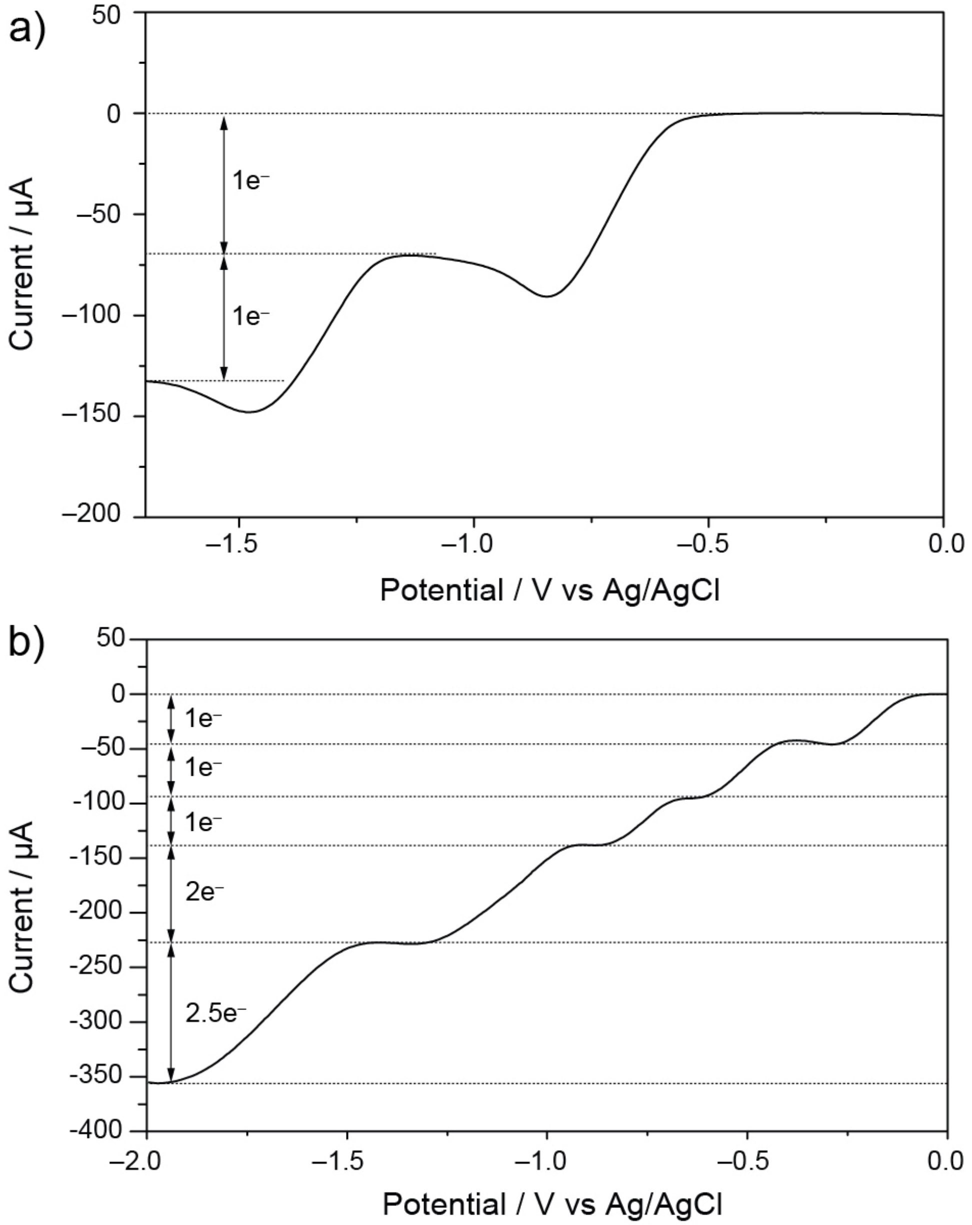

Figure S14. Normal pulse voltammetry of (a) 1.0 mM **XQ** and (b) 1.0 mM **Q-Rot-A** in CH_2Cl_2 solution containing 0.1 M $TBAPF_6$ as the supporting electrolyte

Section G. Computational Studies

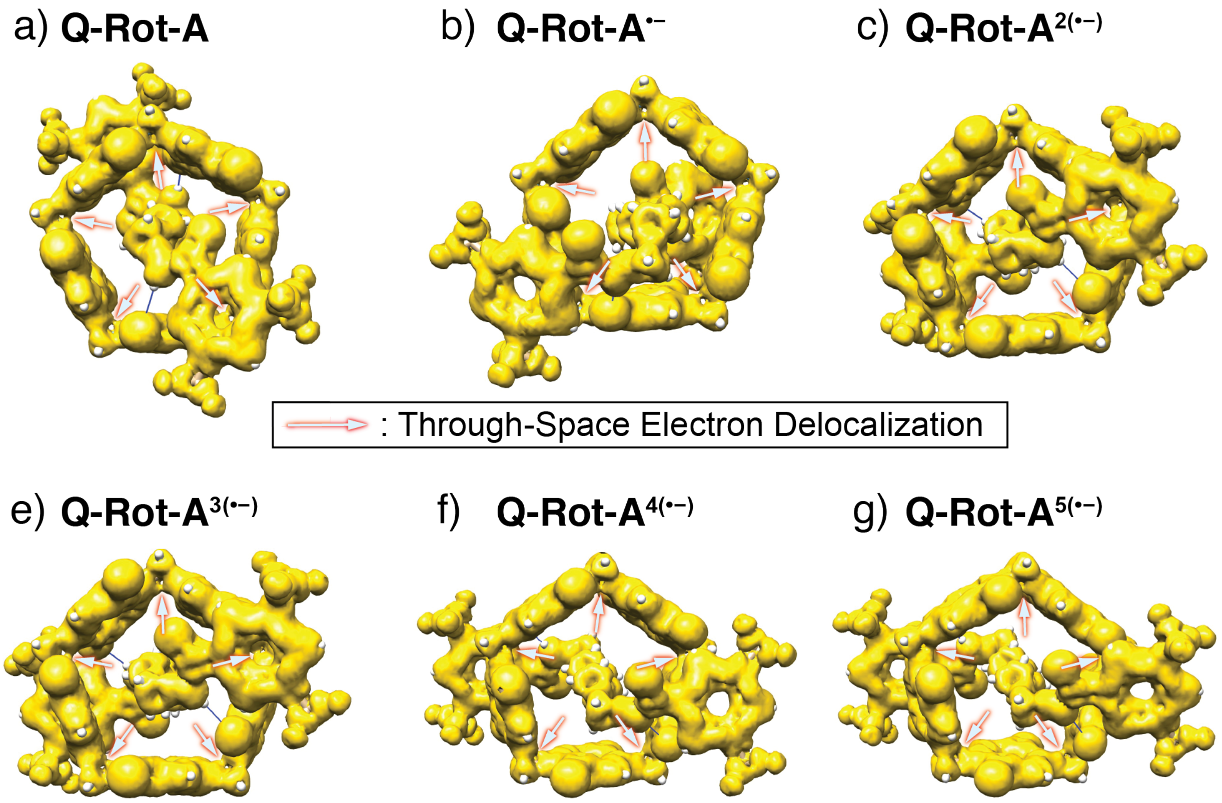

Figure S15. Anisotropy of the induced current density (AICD) calculated at an isovalue of 0.025 for **Q-Rot-A**, **Q-Rot-A**$^{\bullet-}$, **Q-Rot-A**$^{2(\bullet-)}$, **Q-Rot-A**$^{3(\bullet-)}$, **Q-Rot-A**$^{4(\bullet-)}$, and **Q-Rot-A**$^{5(\bullet-)}$

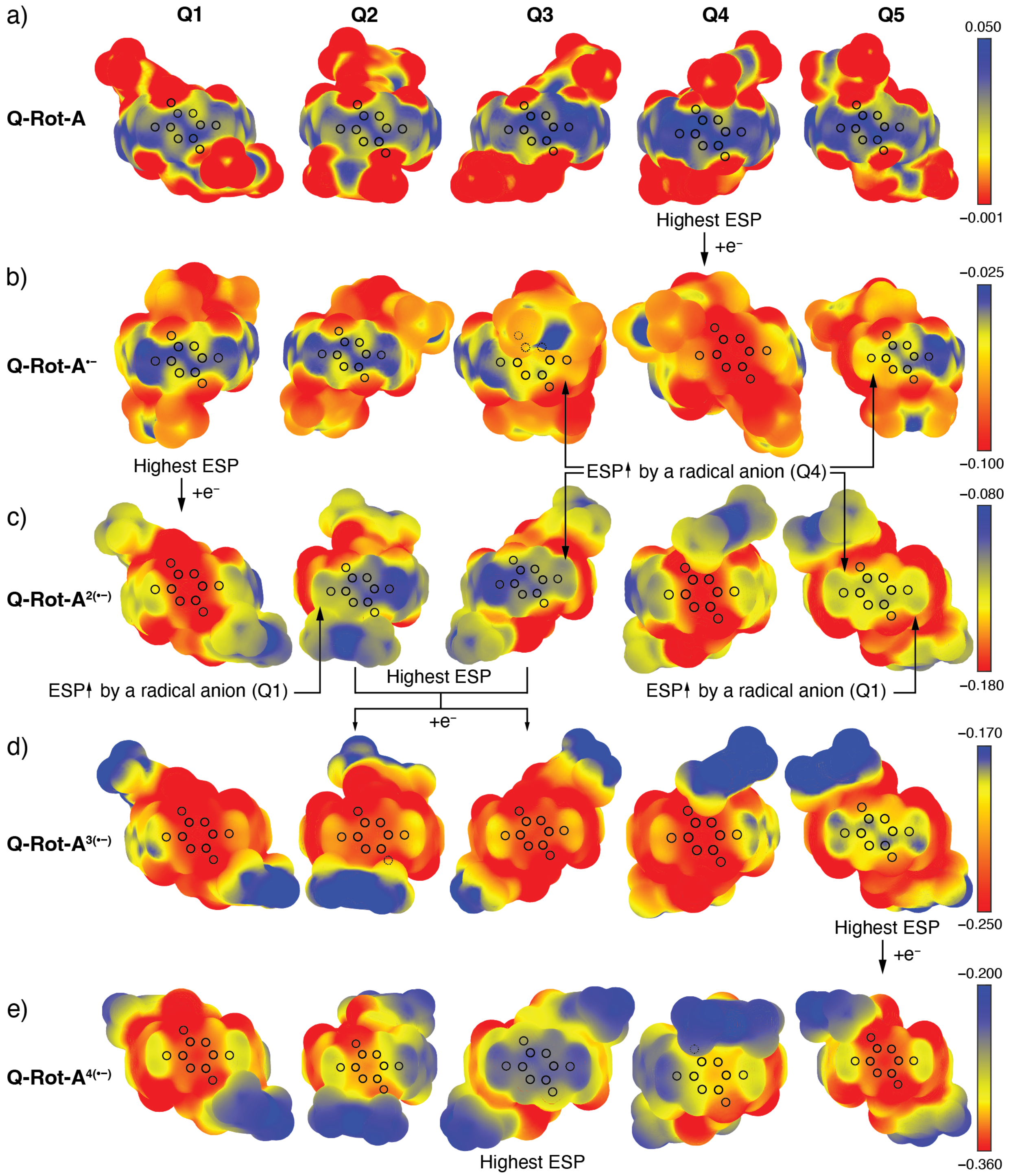

Figure S16. Electrostatic potential (ESP) mapped on the 0.001 au electron density isosurface for **Q-Rot-A**, **Q-Rot-A**$^{\bullet-}$, **Q-Rot-A**$^{2(\bullet-)}$, **Q-Rot-A**$^{3(\bullet-)}$, and **Q-Rot-A**$^{4(\bullet-)}$.

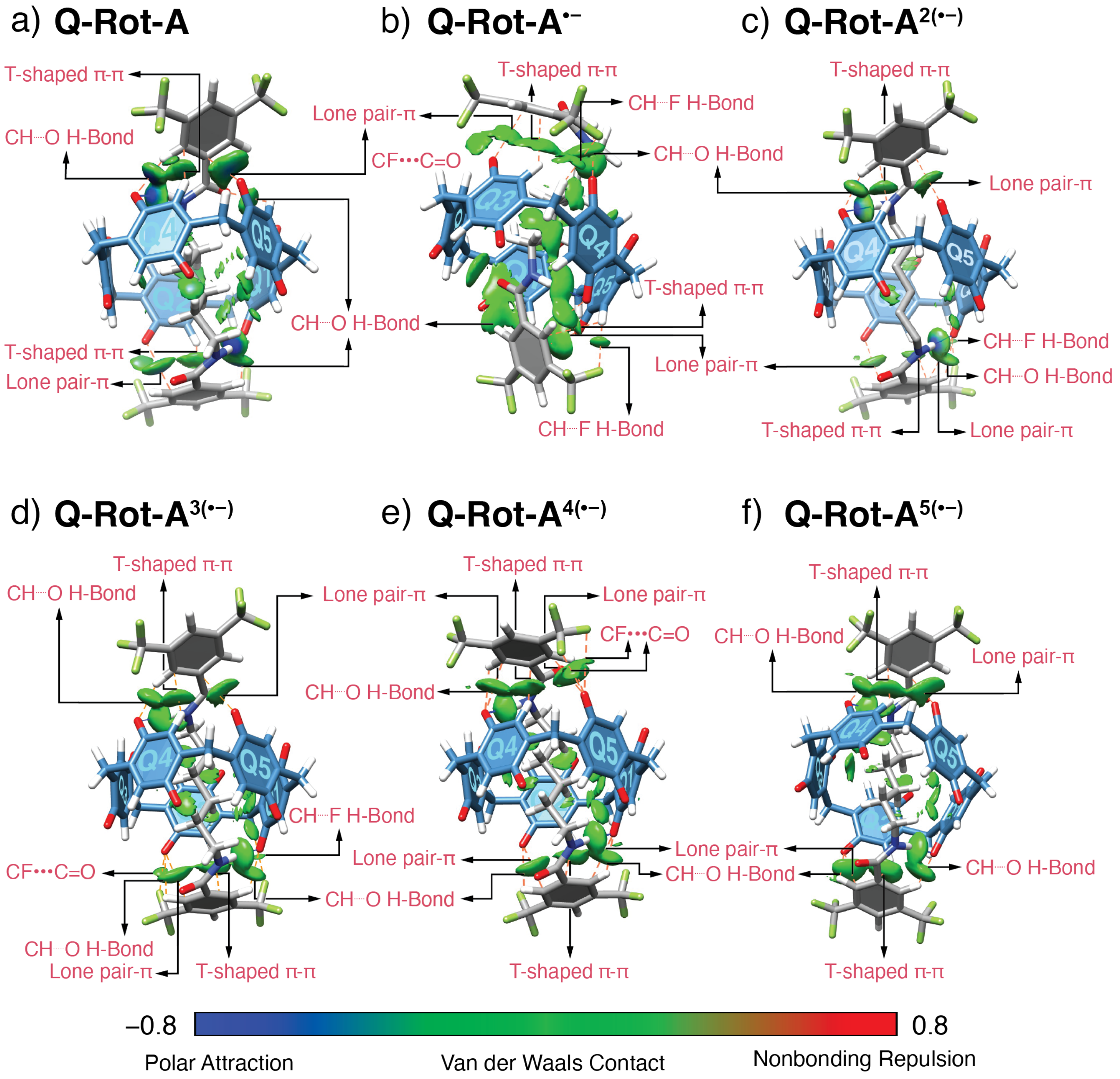

Figure S17. Intercomponent non-colvaent interactions between the dumbbell and ring components of **Q-Rot-A**, **Q-Rot-A$^{\bullet-}$**, **Q-Rot-A$^{2(\bullet-)}$**, **Q-Rot-A$^{3(\bullet-)}$**, **Q-Rot-A$^{4(\bullet-)}$**, and **Q-Rot-A$^{5(\bullet-)}$**, analyzed using the independent gradient model (IGM) at an isovalue of 0.0055. Interactions between the stoppers and the ring are classified as weak hydrogen bonds (CH···O and CH···F), T-shaped π–π interactions, lone pair–π interactions, and multipolar interactions (CF···C=O). These orchestrated interactions stabilize the orientation of the dipole moment pointers (amide groups).

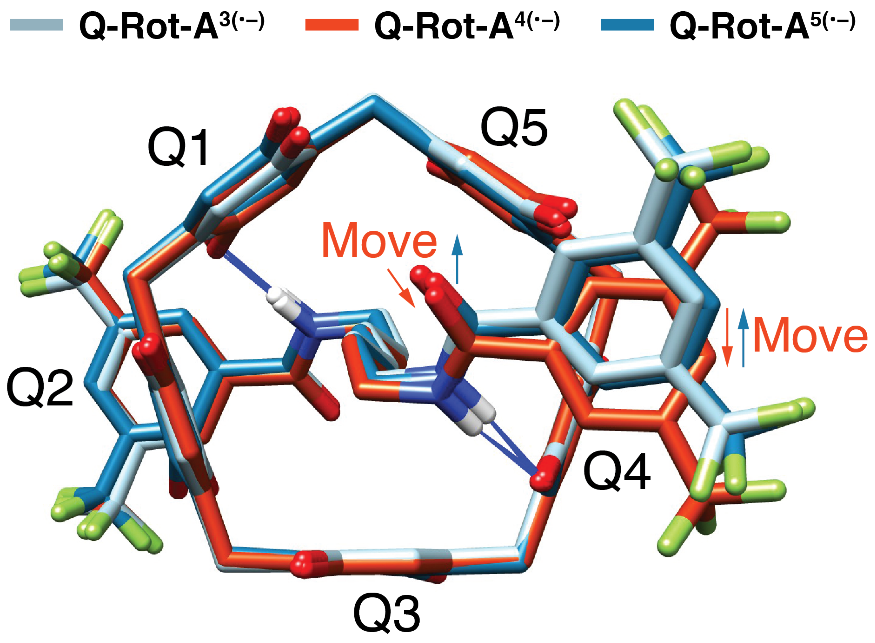

Figure S18. Comparison of optimized structures for **Q-Rot-A**$^{3(\bullet-)}$, **Q-Rot-A**$^{4(\bullet-)}$, and **Q-Rot-A**$^{5(\bullet-)}$. Reduction of Q5 in **Q-Rot-A**$^{3(\bullet-)}$ leads to the displacement of the upper dumbbell component away from Q5$^{\bullet-}$ in **Q-Rot-A**$^{4(\bullet-)}$. Subsequent reduction of Q3 in **Q-Rot-A**$^{4(\bullet-)}$ induces repulsion, causing the upper dumbbell component to move back toward Q5$^{\bullet-}$, resulting in a co-conformation similar to that of **Q-Rot-A**$^{3(\bullet-)}$. The arrow and blue arrows represent the movement of **Q-Rot-A**$^{4(\bullet-)}$, and **Q-Rot-A**$^{5(\bullet-)}$.

Section H. References

(1) Ogoshi, T.; Aoki, T.; Kitajima, K.; Fujinami, S.; Yamagishi, T.-a.; Nakamoto, Y. Facile, Rapid, and High-Yield Synthesis of Pillar[5]arene from Commercially Available Reagents and Its X-ray Crystal Structure. *J. Org. Chem.* **2011**, *76*, 328–331.

(2) Kwon, T.-w.; Song, B.; Nam, K. W.; Stoddart, J. F. Mechanochemical Enhancement of the Structural Stability of Pseudorotaxane Intermediates in the Synthesis of Rotaxanes. *J. Am. Chem. Soc.* **2022**, *144*, 12595–12601.
